# Predicting long-term outcome in anorexia nervosa: a machine learning analysis of brain structure at different stages of weight recovery

**DOI:** 10.1017/S0033291723001861

**Published:** 2023-12

**Authors:** Dominic Arold, Fabio Bernardoni, Daniel Geisler, Arne Doose, Volkan Uen, Ilka Boehm, Veit Roessner, Joseph A. King, Stefan Ehrlich

**Affiliations:** 1 Translational Developmental Neuroscience Section, Division of Psychological and Social Medicine and Developmental Neurosciences, Faculty of Medicine, Technische Universität Dresden, Dresden, Germany; 2 Eating Disorder Research and Treatment Center, Department of Child and Adolescent Psychiatry, Faculty of Medicine, Technische Universität Dresden, Dresden, Germany; 3 Eating Disorder Research and Treatment Center, Department of Child and Adolescent Psychiatry, Faculty of Medicine, Technische Universität Dresden, Dresden, Germany

**Keywords:** Anorexia nervosa, treatment outcome prediction, brain structure, machine learning

## Abstract

**Background:**

Anorexia nervosa (AN) is characterized by sizable, widespread gray matter (GM) reductions in the acutely underweight state. However, evidence for persistent alterations after weight-restoration has been surprisingly scarce despite high relapse rates, frequent transitions to other psychiatric disorders, and generally unfavorable outcome. While most studies investigated brain regions separately (univariate analysis), psychiatric disorders can be conceptualized as brain network disorders characterized by multivariate alterations with only subtle local effects. We tested for persistent multivariate structural brain alterations in weight-restored individuals with a history of AN, investigated their putative biological substrate and relation with 1-year treatment outcome.

**Methods:**

We trained machine learning models on regional GM measures to classify healthy controls (HC) (*N* = 289) from individuals at three stages of AN: underweight patients starting intensive treatment (*N* = 165, used as baseline), patients after partial weight-restoration (*N* = 115), and former patients after stable and full weight-restoration (*N* = 89). Alterations after weight-restoration were related to treatment outcome and characterized both anatomically and functionally.

**Results:**

Patients could be classified from HC when underweight (ROC-AUC = 0.90) but also after partial weight-restoration (ROC-AUC = 0.64). Alterations after partial weight-restoration were more pronounced in patients with worse outcome and were not detected in long-term weight-recovered individuals, i.e. those with favorable outcome. These alterations were more pronounced in regions with greater functional connectivity, not merely explained by body mass index, and even increases in cortical thickness were observed (insula, lateral orbitofrontal, temporal pole).

**Conclusions:**

Analyzing persistent multivariate brain structural alterations after weight-restoration might help to develop personalized interventions after discharge from inpatient treatment.

## Introduction

Anorexia nervosa (AN) is a severe psychiatric disorder characterized by self-starvation and extreme weight loss with typical onset in early adolescence (Treasure et al., 2015) and high mortality rate (Arcelus, Mitchell, Wales, & Nielsen, 2011). Effective interventions are lacking and long-term treatment resources are inadequate in many countries (Berg et al., 2019; Erskine, Whiteford, & Pike, 2016; Solmi et al., 2021). A better understanding of the underlying neurobiology and brain structural alterations in AN might pave the way to better treatments or identify subgroups at increased risk of chronicity.

Sizable and widespread reductions in gray matter (GM) volumes and cortical thickness (CT) have been reported in acutely underweight patients with AN (acAN) relative to healthy controls (HC) (Bahnsen et al., 2022; King, Frank, Thompson, & Ehrlich, 2018; Walton et al., 2022). Previous (longitudinal) studies, including our own, suggested that these reductions are related to body mass index (BMI), i.e. the state of undernutrition, and normalize with weight-restoration (Bahnsen et al., 2022; Bernardoni et al., 2016; Seitz, Herpertz-Dahlmann, & Konrad, 2016). However, some studies found small residual differences in GM morphology, like reduced CT in the right pars orbitalis (Brodrick et al., 2021) or reduced left hippocampus volume (Asami et al., 2022) to persist even after long-term weight-recovery, which might reflect predisposing (trait) factors for AN or consequences of severe illness. Given that former patients often relapse and have high risk for other psychiatric illnesses (Steinhausen et al., 2021), it is somewhat surprising that sMRI studies have struggled to detect morphological alterations after weight-restoration. Importantly, persisting brain alterations might be predictive of future illness course and long-term outcome (Vall & Wade, 2015). The use of biologically objective measurements, such as brain structure, to stratify individuals might enable novel possibilities for personalized precision medicine including improved individualization of prognosis and treatment (Kambeitz-Ilankovic, Koutsouleris, & Upthegrove, 2022). A major limitation of previous sMRI studies in AN is the use of mass univariate analyses, which test each brain region separately for group differences, while psychiatric disorders are theorized to constitute brain network disorders (Fornito, Zalesky, & Breakspear, 2015). Correspondingly, differences in brain network connectivity might be related to structural differences covarying across multiple regions. Under this assumption, machine learning (ML) techniques may be informative as they use multivariate patterns to differentiate groups by combining information from all brain regions. To date, only two studies in comparatively smaller samples have employed ML in AN, showing that underweight (but not weight-recovered) patients can be classified from HC (Lavagnino et al., 2015, 2018).

Our primary aim was to search for multivariate brain structural alterations in participants with AN at two stages of recovery and clarify whether persisting alterations could be of prognostic utility. Specifically, we built ML classifiers to separate HC from (a) underweight patients with AN immediately after admission to an eating disorder program (acAN-TP1) as a baseline, (b) a subset of these patients who achieved partial weight-restoration at the end of intensive treatment (acAN-TP2), and (c) long-term weight-recovered former patients (recAN). We employed regional measures of CT and subcortical volumes as features. In case multivariate alterations were detected in acAN-TP2, we planned to test whether the ML-based risk score as defined below was predictive of 1-year post-admission treatment outcome using the Morgan-Russell outcome assessment scale (Morgan & Hayward, 1988). Predicting outcome after initial weight gain is of particular clinical utility, as clinicians need to make decisions regarding the level and modality of care (e.g. low/high frequency outpatient *v.* day-time) after discharge from intensive treatment (Brockmeyer, Friederich, & Schmidt, 2018). We also note that given the strong dependence of brain alterations in acAN-TP1 on state variables [primarily BMI reduction (Bahnsen et al., 2022; Bernardoni et al., 2018, 2016)], it seems a priori unlikely that these alterations may be reliable biomarkers predictive of outcome above and beyond BMI. Subsequently, we aimed to render our ML results interpretable to point out potential underlying neural mechanisms that might have implications for translational research (Roessner et al., 2021). To this end, we characterized multivariate alterations on which the classifiers relied by (a) identifying the features which contribute most to the classification (explainable AI), (b) contextualizing with network (connectomics) properties, and (c) by exploring whether detected multivariate alterations were temporary or might be trait markers.

## Methods

### Participants

**Figure 1.**
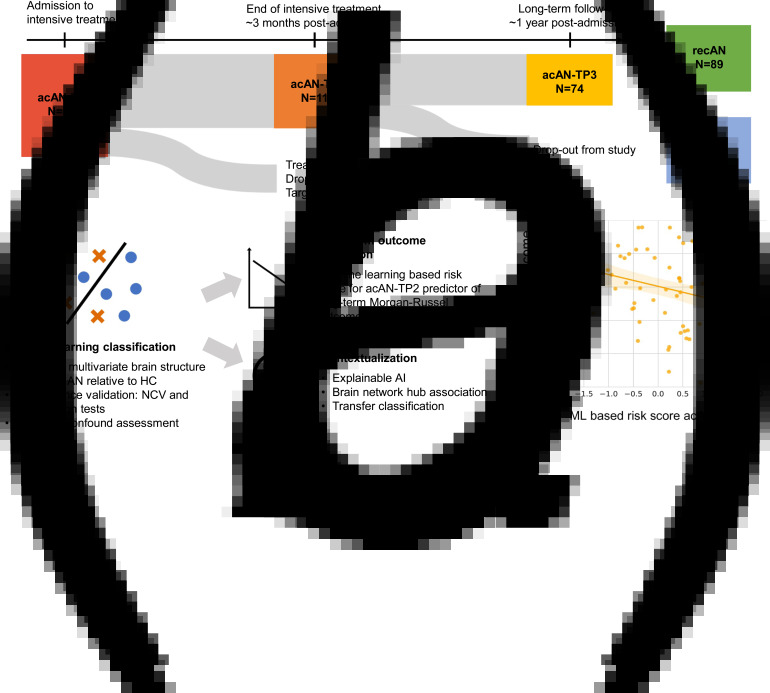
Summary of study design. (a) Included samples in the study. Acute patients with AN were assessed at three time points: within 96 h of treatment initiation (acAN-TP1), after successful weight-restoration treatment occurring approximately 3 months later (acAN-TP2), and at a 1-year follow-up interview (acAN-TP3). Incomplete longitudinal assessment of acute patients occurred due to treatment discontinuation or insufficient BMI gain by the end of treatment, as well as loss of contact for long-term follow-up. Separate cross-sectional samples of long-term weight-recovered former patients (recAN) and healthy control participants (HC) were recruited. Brain MRI scans were acquired in all groups except acAN-TP3. (b) Analysis overview. Structural brain MRI data were processed and used to train machine learning classifiers to differentiate each AN group from HC. The presence of a disorder-related multivariate brain structural pattern in AN was determined through performance estimation using nested cross-validation (NCV), permutation tests, and post-hoc confound assessment. A trained classifier generates a machine learning-based risk score for each individual that provides a measure of how pronounced this pattern is. Given the unclear clinical trajectory of acAN after the initial weight-restoration treatment, we were particularly interested in whether the machine learning based risk score at TP2 was a predictor of long-term clinical outcome at 1-year follow-up (Morgan Russell score at TP3; ‘Long-term outcome prediction’). In an additional line of analyses aimed at interpreting the machine learning results (‘contextualization’), we used explainable AI and other techniques to elucidate the multivariate brain structure pattern found in acAN-TP2 and to investigate its possible biological substrate. (c) Scatter plot for long-term outcome prediction. The machine learning-based risk score in acAN-TP2 was a significant predictor of Morgan Russell outcome at 1-year follow-up, even when adjusting for BMI covariates (see main text for details).

Data from a total of 573 female participants in the greater ongoing Saxonian Anorexia Nervosa Study were analyzed: 302 HC and 271 with AN, see online Supplementary Methods 1.1 for more information on participant recruitment. 68% of these participants were also included in our previous study (Bahnsen et al., 2022). After quality control (see below), the final sample consisted of 658 scans, where participants with AN were included in multiple time-points if scans were available (online Supplementary Fig. S1): 165 acAN scanned within 96 h after beginning nutritional rehabilitation (acAN-TP1; 12–29 years), 115 acAN scanned at the end of an intense treatment program and with a BMI increase of at least 10% (acAN-TP2; 12–25 years), 89 former patients scanned after full and long-term, i.e. at least 6 months, weight-recovery (recAN; 16–30 years), and 289 HC (12–30 years). The inclusion criterion of a 10% increase in BMI between TP1 and TP2 follows established clinical practice and we believe it corresponds to a realistic and clinically-relevant change for inpatients undergoing nutritional rehabilitation. In the actual acAN-TP2 sample, all participants had a BMI increase of ⩾14%. Of all included acAN-TP2, 74 (64%) completed the structured Morgan-Russell interview 1 year after admission to intensive treatment (online Supplementary Table S1). HC participants were recruited according to age in an attempt to obtain independent age-matched case–control samples for each patient group (acAN-TP1/2, recAN). Thus, the pooled HC sample spans the whole age range of participants with AN. Figure 1*a* provides an overview of the samples included this study.

AcAN were admitted to eating disorder programs at the university hospital of the Technische Universität Dresden. AN was diagnosed according to DSM-V criteria using a modified version of the Structured Interview for Anorexia and Bulimia Nervosa [SIAB-EX, (Fichter & Quadflieg, 2001)] and required a BMI<17.5 kg/m^2^ (or below the 10^th^ age percentile, if younger than 15.5 years). To be considered ‘recovered’, former patients had to (a) maintain a BMI>18.5 kg/m^2^ (if older than 18 years) or > 10^th^ age percentile (if younger than 18 years), (b) menstruate, and (c) have not binged, purged, or engaged in restrictive eating patterns for at least 6 months prior to the study. HC were recruited through advertisement among middle school, high school and university students and eating disorders were excluded using the SIAB-EX.

We applied several additional exclusion criteria for all groups beforehand – most importantly, a history of bulimia nervosa or ‘regular’ binge eating, psychotropic medications other than antidepressants (except tricyclic antidepressants and MAO-Inhibitors) within 4 weeks prior to the study, substance abuse and neurologic or medical conditions (online Supplementary Methods 1.1). Participants predominantly identified as ‘European’ (98%; non-European: two acAN, eight HC). Socio-economic status (SES) was determined according to the parental (household) educational level/occupation group (online Supplementary Methods 1.1.1). SES was determined according to the parental (household) educational level/occupation group (Patrick et al., 2004), given that most study participants were adolescent, current students at school, university, or professional training institutions, and still lived with their parents or guardians (online Supplementary Methods 1.1.1).

All AN groups included some participants with at least one comorbid condition (acAN-T1 *N* = 28, acAN-TP2 *N* = 14, recAN *N* = 37; online Supplementary Methods 1.1.1).

### Clinical measures

For acAN patients, treatment outcome 1 year after admission was assessed using the Morgan-Russell assessment schedule (Morgan & Hayward, 1988). This and all other clinical measures in Table 1 were assessed as in previous works [(Bahnsen et al., 2022; Boehm et al., 2016), online Supplementary Methods 1.1.3].

**Table 1.** Sample characteristics

	acAN-TP1 (*N* = 165)	acAN-TP2 (*N* = 115)	acAN-TP2 – acAN-TP1 (*N* = 113)	recAN (*N* = 90)	HC (*N* = 289)
Demographics					
Age (years)	16.49 (3.01) *** [12.20, 29.20]	16.50 (2.31) *** [12.40, 24.60]	0.23 (0.08) *** [0.10, 0.50]	22.18 (3.62) *** [15.50, 29.80]	19.13 (4.37) [12.10, 29.70]
SES	3.50 (1.00) ***	3.50 (1.00) ***	–	4.00 (2.00) n.s.	4.00 (2.00)
IQ	112.01 (11.95) n.s.	112.82 (12.06) n.s.	–	111.96 (10.24) n.s.	112.36 (9.99)
Nutritional status					
BMI (kg/m^2^)	14.75 (1.41) *** [10.82, 17.44]	18.97 (1.11) *** [14.94, 21.32]	4.17 (1.17) *** [2.05, 7.32]	20.97 (1.87) n.s. [17.85, 26.90]	21.15 (2.21) [16.02, 28.66]
BMI-SDS	−3.25 (1.25) ***	−0.76 (0.59) ***	2.43 (0.85) ***	−0.44 (0.60) ***	−0.09 (0.67)
Psychiatric symptom measures					
EDI-2	212.59 (46.49) ***	195.57 (47.39) ***	−13.64 (36.5) ***	161.86 (45.92) ***	135.17 (27.00)
BDI-2	23.42 (11.15) ***	14.74 (10.85) ***	−8.98 (9.70) ***	8.05 (8.37) ***	4.39 (4.92)

Sample size N and mean (s.d.) [range] values for each group and for longitudinal changes within the acAN group. Categorical SES values are shown as median (interquartile range). IQ was assessed in both acAN groups at admission to treatment. Each AN group was contrasted to HC using independent samples *t* tests (columns 1,2,4). The significance of longitudinal differences between acAN-TP1 and acAN-TP2 was assessed through dependent samples *t* tests (column 3). For variables showing significant deviations from normality, a corresponding nonparametric test was used instead (Mann–Whitney U or Wilcoxon; for SES additionally with continuity correction). *p < 0.05, **p < 0.01, ***p < 0.001, n.s. not significant. In acAN-TP1, the time since onset of AN was 14.22 (s.d. = 18.38) months on average, 140 participants (84.8%) were of the restrictive and 21 (12.7%) of the binge/purge subtype. The average duration of (partial) weight-restoration treatment in the acAN-TP2 sample was 2.81 (s.d. = 0.99) months, during which BMI increased by an average of 28.6 (s.d. = 9.6) %. Within recAN, 67 participants (72.8%) were restrictive and 25 (27.2%) binge/purge. The time between acAN-TP1 and acAN-TP2 scans was 2.78 (s.d. = 0.87) months on average and the time since weight-recovery for recAN was 53.54 (s.d. = 38.79) months on average and for almost all participants at least 12 months. Abbreviations: AcAN, participants with acute anorexia nervosa in the acutely underweight state (TP1) or after short-term weight-restoration (TP2); RecAN, long-term weight-recovered participants with a history of AN; HC, healthy control participants; SES, socio-economic status; IQ, intelligence quotient; BMI, body mass index; BMI-SDS, body mass index standard deviation score; EDI-2, averaged score comprising the core subscales drive for thinness, body dissatisfaction, and bulimia of Eating Disorder Inventory-2; BDI-II, Beck Depression Inventory-II.

### MRI acquisition and processing

All participants underwent MRI scanning between 8 and 9 a.m. following an overnight fast. Scanning procedures, Freesurfer preprocessing, and quality control (41 scan exclusions from a total of 699: 11 acAN-TP1, 14 acAN-TP2, 3 recAN, 13 HC) were identical to our previous study (Bahnsen et al., 2022) and are described in detail in online Supplementary Methods 1.2.

### Machine learning classification

We trained classifiers to differentiate (a) acAN-TP1, (b) acAN-TP2, or (c) recAN *v.* HC, respectively, based on 110 input features consisting of CT measures according to the Desikan-Killiany atlas (Desikan et al., 2006) and subcortical volumes. The analysis pipeline consisted of three steps. First, we used *cross-validated confound regression* (Snoek, Miletić, & Scholte, 2019) to subtract the confounding effects of age from all features and the confounding effects of estimated intracranial volume (eTIV) solely from volumetric features. Importantly, effect estimation was performed exclusively on HC training data to avoid subtracting disease related effects (Dukart, Schroeter, Mueller, & The Alzheimer' Disease Neuroimaging Initiative, 2011). Confounding effects were subsequently subtracted in all participants (both HC and AN, and both in the training and test sets). While this method, like other de-confounding methods, might fail to completely remove confounding information, potentially leading to biased model performance, we tested post-hoc whether the obtained classifiers were relying on information unrelated to confounds for the classification (see below). Second, we applied PCA for dimensionality reduction. Finally, classification was performed by a linear L2-regularized SVM. However, to evaluate whether nonlinear patterns in the data could enhance classification performance, we utilized pipelines that employed a neural network instead of the linear SVM (online Supplementary Methods 1.3). Both classifier types generate binary class predictions by thresholding their continuous output, which we refer to as *ML-based risk score*. We jointly optimized the number of PCA components and the SVM hyperparameters via repeated stratified 10-fold cross-validated grid search (online Supplementary Table S2). Specifically, this means that all model parameters were optimized only on training data within cross-validation to avoid information leakage. To consider class imbalances, we used precision (positive predictive value)-recall (sensitivity) area under the curve (PR-AUC) as optimization and performance metric (Saito & Rehmsmeier, 2015) and weighted the cost of misclassification for a participant by the inverse of her group frequency. In addition to PR-AUC, we also report area under the receiver operator characteristic curve (ROC-AUC) to allow comparisons between classifiers aimed to differentiate patients with AN at different time points from HC, which were trained on data sets with different class ratios. To obtain unbiased model performance estimates [specifically since our optimization procedure involved the optimization of hyperparameters (online Supplementary Methods 1.4, Table S2)], we applied (to the whole model pipeline) nested cross-validation which partitions the dataset into training, validation, and test sets (online Supplementary Methods 1.5, Fig. S2). Training and evaluation of ML pipelines was done in Python *v.* 3.6.12 using the Scikit-learn library *v.* 0.23.2 (Pedregosa et al., 2011).

### Model analysis

Based on ML results, we tested whether the ML-based risk score for acAN-TP2 was predictive of long-term outcome. An additional line of analysis served to enable interpretation of ML results (Fig. 1*b*).

#### Confound assessment

To assess the role of confounding variables (e.g. age, eTIV) on performance estimates despite subtraction of linear effects, we examined the extent to which the predictive ability of the ML-based risk score could be attributed to these confounds. To this end, we computed post-hoc shared and exclusive deviance explained by confounding variables and ML-based risk score when predicting group membership (Dinga, 2020). Permutation resampling was performed to compute significance levels for deviances and classification performances [(Ojala & Garriga, 2010), online Supplementary Methods 1.6, 1.7].

#### Long-term outcome prediction

For acAN-TP2 with complete outcome data (*N* = 74), we tested whether potential multivariate structural alterations were related to long-term outcome. To this end, we built GLMs with Morgan-Russell outcome score as dependent variable and the ML-based risk score as independent variable. We considered a GLM with no additional covariate, then three additional GLMs with (i) current BMI standard deviation score (BMI-SDS; online Supplementary Methods 1.1.1), or (ii) BMI-SDS increase since admission to treatment, or (iii) the presence of comorbid psychiatric disorders as additional covariates (online Supplementary Methods 1.8).

#### Explainable AI

We utilized the concept of feature importance to estimate the relevance each measure of brain structure (feature) had for the classifier's prediction, i.e. how much information useful to differentiate AN from HC each feature provided. We measured feature importance as a model's activation pattern (Haufe et al., 2014). In our case of linear models, this was effectively done by determining the correlation coefficient of each feature with the ML-based risk score. The positive/negative sign of feature importance values indicates whether a higher/lower feature value was characteristic for AN. For each feature, we further assessed a measure of *reliability* as the fraction of subsamples of the dataset where its feature importance was significant [(Nogueira & Brown, 2016), online Supplementary Methods 1.9]. To further improve the interpretability of our results, we additionally applied the method of permutation importance (Breiman, 2001) to identify the subset of important features which contain the highest amount of unique information useful for classification which is not also present in other (correlated) features (online Supplementary Methods 1.9).

#### Connectome contextualization

We also tested profiles of feature importance for associations with normative structural and functional connectome data from a public healthy reference sample [Human Connectome Project (HCP), (Toga, Clark, Thompson, Shattuck, & Van Horn, 2012)] to understand patterns of (sub-) cortical alteration in the context of macro-scale network organization using the ENIGMA Toolbox [(Larivière et al., 2021), online Supplementary Methods 1.10]. Specifically, structural and functional connectivity matrices from the HCP data were used to compute weighted degree centrality, i.e. the number of ties that a connectivity node has. This served as a measure of ‘network centrality’, which indicates how highly a brain region is connected to the rest of the brain (Bullmore & Sporns, 2009). Subsequently, Pearson's coefficients between network centrality (assessed in HCP data) and feature importance profiles (assessed in our sample) were computed. This method assumes that regions that are identified as network hubs in HC will also exhibit this characteristic in individuals with psychiatric conditions (Larivière et al., 2021).

#### Effect of nutritional status and disease state

To clarify the role of BMI on classifier performance and gain insight as to whether the multivariate alterations detected were state or potential trait-markers, we applied the confound assessment method used to control for age and eTIV (see above) to BMI-SDS as well (Dinga, 2020)**.** Further, similar to recent work in ADHD (Zhang-James et al., 2021), we explored whether multivariate alterations detected for AN groups at different illness stages express temporal continuity. Specifically, we applied a model trained on a certain classification task (e.g. acAN-TP2 *v.* HC) to another classification task (e.g. acAN-TP1 *v.* HC). Importantly, no scans belonging to the same participant (taken at different time points) were used both in the training and test sets (transfer classification, online Supplementary Methods 1.11).

## Results

### Clinical characteristics

Sample demographic and clinical characteristics are summarized in Table 1 and online Supplementary Table S1.

### Machine learning classification

**Figure 2.**
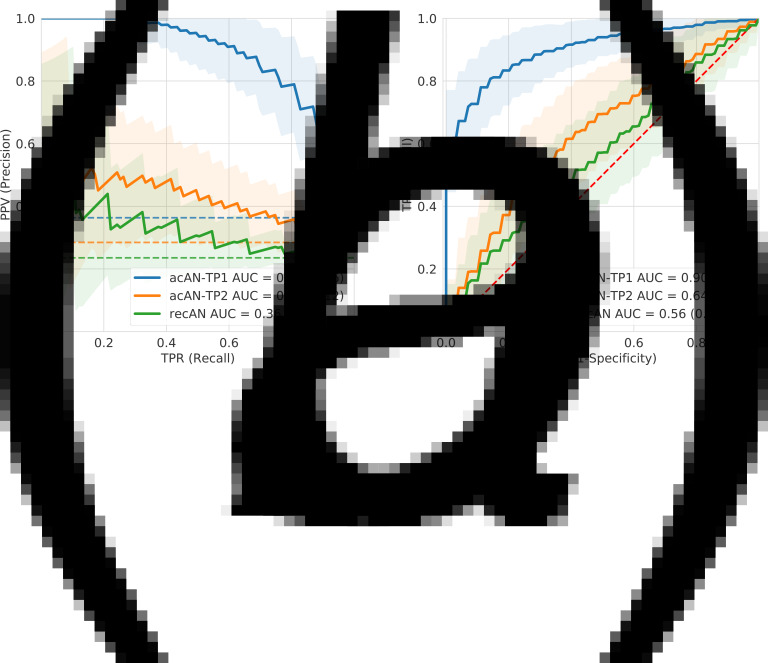
Visual comparison of the test performances achieved by the support vector machine classifiers. Test performance curves were estimated using (10 times repeated, 10-fold) nested cross-validation for acAN-TP1 (blue), acAN-TP2 (yellow), and recAN (green) *v.* HC classifications. The Precision-Recall (a) and corresponding receiver operating characteristic (ROC) (b) curves show test performance averages and s.d. ranges and provide an estimate for the performance of the model selection procedure (online Supplementary Methods 1.4). The dashed lines represent chance performance. Precision-Recall AUC was optimized during training. Since Precision is sensitive to group sizes, Precision-Recall curves are not comparable across classification tasks with different AN groups. Therefore, also the corresponding ROCs are shown. Permutation tests of the corresponding AUCs showed clear above-chance classification for acAN-TP1 and acAN-TP2 but not for recAN (online Supplementary Methods 1.6, Fig. S4).

As expected, the highest test performance was achieved for classification of acAN-TP1 *v.* HC (PR-AUC = 88.7%, ROC-AUC = 90.2%), followed by acAN-TP2 *v.* HC (PR-AUC = 45.5%, ROC-AUC = 63.6%) and recAN *v.* HC (PR-AUC = 35.9%, ROC-AUC = 56.3%) (Fig. 2, online Supplementary Results 2.1, Fig. S3). Permutation tests confirmed that performances were above chance for both acAN time points, but not for recAN (online Supplementary Fig. S4), both when looking at PR-AUC (acAN-TP1: *p* < 0.001, acAN-TP2: *p* = 0.001, recAN: *p* = 0.091) and at ROC-AUC (acAN-TP1: *p* < 0.001, acAN-TP2: *p* = 0.001, recAN: *p* = 0.055). Additionally, for both acAN models, but not for the recAN model, the explained deviance independent of age and eTIV was significantly positive (Table 2). Replacing the SVM with a neural network in the model pipeline did not improve performance or alter key findings (online Supplementary Methods 2.2, Fig. S5).

**Table 2.** Deviance explained by ML-based risk scores and confounds

	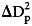	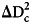	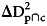	Confounds
acAN-TP1 *v.* HC	0.44***	0.08	0.04***	Age, eTIV
acAN-TP2 *v.* HC	0.07**	0.14	0.00 n.s.	Age, eTIV
acAN-TP2 *v.* HC	0.05*	0.32	0.02*	Age, eTIV, BMI-SDS
recAN *v.* HC	0.04 n.s.	0.12	0.01 n.s.	Age, eTIV

For each classification model and set of confounds (last column) we report the proportion of deviance explained exclusively by model predictions 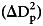, exclusively by confounds 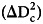, or by both model predictions and confounds 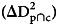 (Dinga, 2020). Explained deviance of model predictions beyond confounds was significant at both acAN time points. Significance of these estimates was assessed using permutation tests. *p < 0.05, **p < 0.01, ***p < 0.001, n.s. not significant.

### Long-term outcome prediction

The acAN-TP2 ML-based risk score significantly predicted (*p* = 0.015, *R*^2^ = 0.065) Morgan-Russell Outcome score 1 year after admission to the treatment program [*N* = 74, average age = 17.19 years (s.d. = 1.89), average BMI = 18.95 kg/m^2^ (s.d. = 1.69)], suggesting that acAN-TP2 with more pronounced AN-specific multivariate alterations had a worse long-term outcome. This main result is also visualized in Fig. 1*c*. The acAN-TP2 ML-based risk score was not significantly correlated with known and clinically relevant outcome predictors like current BMI-SDS and BMI-SDS change since admission to treatment (*r* = −0.14, *p* = 0.15 and *r* = −0.06, *p* = 0.52), nor did it differ between patients with and without psychiatric comorbidity (Mann–Whitney *U* test *p* = 0.12). The ML-based risk score remained a significant predictor when adding either of these variables as a covariate to the regression model (BMI-SDS *p* = 0.025, BMI-SDS increase *p* = 0.016, and binary comorbidity flag *p* = 0.015).

### Explainable AI

**Figure 3.**
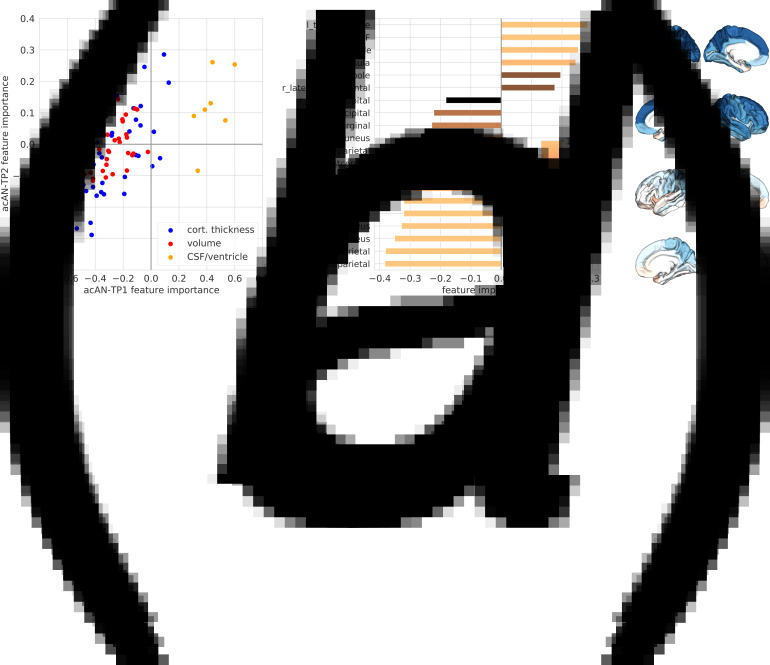
Feature importance analyses. The explainable AI results show the importance of each measure of brain structure (feature) for classification. Feature importance was defined as the Pearson correlation coefficient between each feature and the machine learning-based risk score (Haufe et al., 2014). More positive/negative values indicate that a larger/smaller value for a feature is characteristic of AN. (a) All feature importance values for the acAN-TP1 model (*x*-axis) compared to values for the acAN-TP2 model (*y*-axis). Feature importances for measures of cortical thickness (CT), volumes of subcortical gray matter (GM) regions, and cerebrospinal fluid (CSF) spaces are shown in blue, red, and orange, respectively. While most features are highly relevant for the classification acAN-TP1 *v.* HC, subcortical GM volumes lose relevance compared with CT and CSF spaces for the acAN-TP2 *v.* HC classification. (b) Features ranked by importance for the acAN-TP2 *v.* HC classification. Only features whose importance was significant after applying a Bonferroni correction for multiple comparisons are listed. The color code illustrates which were the most reliable features for classification. The reliability value is the percentage of cases in which the feature importance is significant across models trained on different subsamples of the entire data set (online Supplementary Methods 1.9). Features with a reliability >0.9 were the CT of superiorparietal, inferiorparietal, paracentral, left cuneus, and left postcentral regions (negatively signed importance), as well as CT of the insula and left temporal pole, and volumes of 3^rd^ ventricle and total CSF space (positively signed importance). (c), (d) The same feature importance values for the acAN-TP1/TP2 model plotted on the surface of the standard average brain (Larivière et al., 2021). The color code illustrates the magnitude of negatively (blue) and positively (red) signed feature importance.

Most features had a high importance in the acAN-TP1 model (Fig. 3*a*, online Supplementary Fig. S6), whereas the acAN-TP2 model mainly relied on measures of CT and fluid space volumes rather than volumes of subcortical GM regions (Fig. 3*b*). Nevertheless, we observed a strong correlation between acAN-TP1 and acAN-TP2 feature importance values (*r* = 0.70, *p* < 0.001). Consistently, only features with high importance also show high reliability values for the acAN-TP2 model (Fig. 3*b*) which indicates stability of the found multivariate pattern. While most features had only a moderate reliability for the acAN-TP2 model, several features had a reliability above 0.9 (online Supplementary Fig. S7). Within this more conservative subset of highly reliable features were several CT-based features with large negative importance, i.e. participants with lower CT in these brain regions had a higher probability of being classified into the AN group (Fig. 3*d*). The same was observed in the acAN-TP1 model which, however, based its decision on a considerably larger set of features with negatively signed importance, including hemispheric CT averages (Fig. 3*c*, online Supplementary Fig. S8). Conversely, cerebrospinal fluid (CSF) spaces had positively signed importance values both in the acAN-TP1 and in the acAN-TP2 model. Interestingly, left insula, right lateral orbitofrontal, and bilateral temporal poles CT had positively signed importance and high reliability in the acAN-TP2 model (Fig. 3*b*), but were irrelevant in the acAN-TP1 model. These findings remained unaltered when using a neural network instead of an SVM for classification (online Supplementary Fig. S9). Only a subset of high positive importance features (Fig. 3*b*) additionally showed a high permutation importance (bilateral insula, right lateral orbitofrontal CT), indicating that they provided unique classification information (online Supplementary Results 2.3, Fig. S10).

### Connectome contextualization

The regional CT feature importance profile derived from our acAN-TP2 model correlated with functional cortical network centrality based on HCP data (*r* = −0.65, *p* = 0.006, online Supplementary Fig. S11). In other words, cortical regions whose thickness had high negatively signed importance for classification can be characterized as hubs, i.e. regions with high network centrality. This was also the case for the feature importance profile from the acAN-TP1 model for functional and structural cortical network centrality (*r* = −0.67, *p* < 0.001 and *r* = −0.43, *p* = 0.001, online Supplementary Fig. S12).

### Effect of nutritional status and disease state

When adding BMI-SDS to age and eTIV for confound analysis of the acAN-TP2 *v.* HC classification, the shared and model exclusive deviance were reduced, but still significant (Table 2). Further, the acAN-TP2 model was successful in classifying acAN-TP1 *v.* HC (transfer classification, online Supplementary Figs S13, S14), but the reverse was not true, in accordance with the observation that a large set of features can be exploited by the acAN-TP1 model (Fig. 3*a*, online Supplementary Fig. S9), while the acAN-TP2 model relied on a more specific pattern of a reduced subset of features.

## Discussion

We applied ML methods to classify individuals with a history of AN at two stages of recovery from HC using sMRI measures. Most importantly, and in contrast to inconclusive evidence in previous univariate studies, successful classification (ROC AUC = 0.64, estimated with nested cross-validation and significantly above-chance *p* = 0.001) of partially weight-restored patients (acAN-TP2) indicated multivariate structural differences relative to HC at this early stage of weight-recovery. Underlining the potential clinical utility of this finding, the ML-based risk score was a predictor of future outcome. Explainable AI analyses in the form of *feature importance* revealed that classification of partially weight-restored patients relied on reductions in CT and GM volumes, and similarly to underweight patients, these were more pronounced in hubs, i.e. regions with greater functional connectivity according to normative brain connectome organization in healthy individuals. However, in contrast to acutely underweight patients, classification of partially weight-restored patients could not be attributed to nutritional (BMI) status, and it even relied on increased CT in some regions (insula, lateral orbitofrontal, and temporal pole). These results replicated when excluding adults from the analysis, suggesting that they primarily apply to adolescents (online Supplementary Results 2.4). Classification of underweight patients (acAN-TP1) from HC served as a baseline and resulted, as expected, in high performance (ROC AUC = 0.90). In contrast, classifying long-term weight-recovered former patients (recAN) from HC was not possible despite the increased sensitivity of the multivariate approach used. Together, these latter results mirror those from previous studies using standard mass-univariate approaches indicating sizable and widespread reductions of CT and subcortical GM volume in acutely underweight individuals with AN, but relative normalization following long-term weight-recovery (Bahnsen et al., 2022). Our study is the first in AN that provides an estimate of the performance achieved using nested cross-validation and therefore serves as a valuable benchmark for this rapidly developing field of research (Bracké et al., 2023; Walter et al., 2019).

While our results are in line with previous findings in acAN and recAN (King et al., 2018), the successful classification of acAN-TP2 from HC stands in contrast to previous univariate studies that found no differences in CT or GM volumes after short-term weight-restoration (Bahnsen et al., 2022; Bernardoni et al., 2016). This might be owed to the greater sensitivity of the multivariate methods, which relied on a large set of measures from anatomical regions distributed across the brain. Of note, rigorous permutation tests confirmed that classification performance was significantly above chance, and did not rely on accidental group differences in age or intracranial volume.

Suggesting possible implications for clinical practice, the acAN-TP2 ML-based risk score was predictive of individual treatment outcome (Morgan Russell score), also when controlling for BMI-SDS at discharge or BMI-SDS increase during therapy, which are considered relatively established objective outcome predictors (Boehm et al., 2016; Vall & Wade, 2015). In other words, the multivariate structural brain alterations in acAN-TP2 on which the classifier relied were most expressed in individuals with an unfavorable long-term outcome. Previous works reported cerebellar GM volume of patients with AN at admission to treatment to be predictive of BMI at discharge (Milos et al., 2021) and 1-year follow-up (Seitz et al., 2015). In contrast, here we detected a multivariate pattern of brain structure alterations in partially weight-restored patients which did not involve cerebellar measures – see below. Furthermore, we used the Morgan-Russell interview as an outcome measure, which covers both physiological (including BMI status at 1-year follow-up) as well as psychological recovery.

Given that previously studied objective treatment outcome predictors mostly relied on BMI (Vall & Wade, 2015), we focused on the biological substrate of the identified multivariate alterations and its relationship to BMI status. To this end, we computed each model's feature importance values, which provide for each anatomical measure an interpretable relevance score for a given classification (Haufe et al., 2014). Feature importance values for the acAN-TP1 and acAN-TP2 models correlated and regions associated with negative importance were functional hubs in both models, suggesting that the alterations revealed in acAN-TP2 might reflect incomplete normalization. A recent study in acutely underweight patients with AN also found more severe CT reductions in network hubs, and argued it might be related to their higher metabolic demand (Bahnsen et al., 2022). Hubs are vital nodes in normative brain network functioning and pathological impairment of those has been hypothesized to possibly cause more severe maladaptive brain network re-organization and thus lead to worse prognosis (Fornito et al., 2015). Thus, the multivariate alterations found in acAN-TP2 could constitute brain structural consequences of recent undernutrition, but may nonetheless affect long-term outcome despite considerable BMI normalization. Confirming the similarity of the multivariate alterations from another perspective, the acAN-TP2 model could also be used to classify acAN-TP1 from HC (ROC-AUC = 0.81). However, while not salient in acAN-TP1, acAN-TP2 classification was also characterized by higher CT in left insula, right orbitofrontal cortex, and bilateral temporal poles. The former two regions and right insula showed the highest permutation importance, indicating that they might constitute distinguished and regionally specific alterations in patients with AN. Research on patients with AN following partial weight-restoration is scarce and different protocols regarding recovery status were used (King et al., 2018). Our findings align with those of three previous studies on patients who received intensive treatment for about 2 weeks (Frank, Shott, Hagman, & Mittal, 2013a; Frank, Shott, Hagman, & Yang, 2013b; Lavagnino et al., 2018). However, a recent meta-analysis (using the common linear regression approach) did not substantiate significant regional increases in AN patients following partial weight-restoration relative to HC (Walton et al., 2022). The insula, relevant in processing interoceptive information (Simmons et al., 2013), was repeatedly shown to be involved in AN psychopathology (Jacquemot & Park, 2020). Speculatively, since insula and orbitofrontal cortex are important for taste perception and (food) reward valuation (Frank, Shott, & DeGuzman, 2019; Suzuki, Cross, & O'Doherty, 2017), CT in these regions might be higher in individuals with AN already premorbidly, but relatively suppressed in the underweight state. The relevance of these two regions has been discussed previously based on evidence suggestive of altered structural connectivity between them in individuals with AN (Frank, Shott, Riederer, & Pryor, 2016; Shott, Pryor, Yang, & Frank, 2016). Otherwise, increased insula CT might develop during weight-restoration, e.g. through lipid dysregulation occurring during rapid refeeding (Tam et al., 2021). Further evidence demonstrating that the multivariate alterations found after partial weight-restoration do not merely reflect incomplete normalization, rigorous post-hoc analysis based on permutation tests revealed that the acAN-TP2 model could classify above and beyond BMI-SDS alone.

Our study design cannot discriminate whether these alterations did already exist premorbidly or represent sequelae of the illness/treatment. However, consistent with the finding that they are more pronounced in patients with a worse outcome, no alterations were detected in our sample of well-recovered former patients, who had a comparatively favorable outcome, met strict inclusion criteria, and were weight-recovered for 53.5 (s.d. = 38.8) months on average, neither by transferring the acAN-TP2 classifier to this sample, nor by training a dedicated recAN classifier. Longitudinal studies following patients for longer periods of time (>1 years) are needed for further insights regarding this question.

### Limitations

This study comes with some limitations. First, our single-site analysis based on rather young participants who mostly self-identified as European may not generalize to adult or chronically ill patients, or to patients from other treatment centers and different ethnicities. Furthermore, due to our exclusion criteria, AN groups included fewer participants with comorbidities than would be expected from epidemiological data. While this group is of special interest for improved outcome prediction, future studies should attempt to include participants with more severe psychiatric load. However, we rigorously tested generalizability of our results to unseen participants not used for optimizing the classifier using nested cross-validation, performed permutation tests to determine whether classifier performances were significantly above chance, and assessed potential effects of confounding variables. Second, we relied on derived neuroimaging phenotypes based on the Desikan-Killiany parcellation to classify participants with AN from HC. Higher sample sizes of similar quality might enable e.g. application of deep learning models to raw imaging data to potentially discover even more refined nonlinear multivariate patterns unconstrained by an a priori choice of parcellation. Therefore, our inability to classify long-term recovered former patients with a favorable outcome from HC should not be interpreted as complete absence of structural differences, which could be predisposing factors or scars. Third, while obtaining and testing longitudinal trajectories of structural brain changes in AN would be desirable, it was not feasible in the scope of the current study.

### Implications for clinical use

In summary, we identified a multivariate pattern of subtle regional brain structural alterations in short-term weight-restored patients with AN, which was largely unrelated to current nutritional status and might be predictive of long-term treatment outcome and thus complement other predictors of prognosis such as BMI status. If our results are reproduced in independent samples, our approach might be a foundational step for future research aimed toward clinical translation. Specifically, sMRI scans at the end of intensive weight rehabilitation treatment could be performed from which a classifier (trained on an expanding set of past patients scans) would deliver a ML-based risk score for new individual patients. This score, indicating brain health, might be helpful in combination with other predictors to estimate future outcome and assess whether the patient may need more comprehensive interventions, e.g. home-treatment (Herpertz-Dahlmann et al., 2021), after discharge from intensive treatment. Importantly, however, in light of the above limitations, our results should not be seen as a ready-to-use algorithm, but as a starting point for more research to build and evaluate such an instrument.

## Supporting information

Arold et al. supplementary material Arold et al. supplementary material

## Data Availability

The data that support the findings of this study are available from the corresponding author, s.e., upon reasonable request. Our analysis code is publicly available (https://doi.org/10.17605/OSF.IO/MCJP6).

## Supplementary material

The supplementary material for this article can be found at https://doi.org/10.1017/S0033291723001861.
