# Supplementary material for: Predicting long-term outcome in anorexia nervosa: a machine learning analysis of brain structure at different stages of weight recovery
Source: Psychol Med. 2023 Aug 9;53(16):7827–36. doi: 10.1017/S0033291723001861 (PMC10758339; doi:10.1017/S0033291723001861)
Supplement: Arold et al. supplementary material [file S0033291723001861sup001.docx]

Supplementary Information

**Predicting long-term outcome in anorexia nervosa: a machine learning analysis of brain structure at different stages of weight recovery**

Dominic Arold^1^, Fabio Bernardoni^1^, Daniel Geisler^1^, Arne Doose^1^, Volkan Uen^1^, Ilka Boehm^1^, Veit Roessner^2^, Joseph A. King^1^, Stefan Ehrlich^1,3^

^1^Division of Psychological and Social Medicine and Developmental Neurosciences, Faculty of Medicine, Technische Universität Dresden, Dresden, Germany

^2^Department of Child and Adolescent Psychiatry, Faculty of Medicine, University Hospital C. G. Carus, Technische Universität Dresden, Dresden, Germany

^3^Translational Developmental Neuroscience Section, Eating Disorder Research and Treatment Center, Department of Child and Adolescent Psychiatry, Faculty of Medicine, Technische Universität Dresden, Dresden, Germany

**Supplementary methods.**

**Supplementary results.**

**Table S1.** Group comparison for partially weight restored patients

**Table S2.** Optimal hyperparameters for SVM models

**Figure S1.** Sample overlaps

**Figure S2.** Nested cross-validation method

**Figure S3.** Nested cross-validation results

**Figure S4.** Permutation test distribution for classification performance

**Figure S5.** Classification performance of neural network model

**Figure S6.** Feature importance for acAN-TP1 vs HC classification

**Figure S7.** Reliability values for the acAN-TP2 vs HC classification

**Figure S8.** Reliability values for the acAN-TP1 vs HC classification

**Figure S9.** Results for neural network model

**Figure S10.** Permutation importance

**Figure S11.** Connectivity contextualization for acAN-TP2

**Figure S12.** Connectivity contextualization for acAN-TP1

**Figure S13.** Transfer classification

**Figure S14.** Transfer classification for neural network model

**Supplementary references.**

## Supplementary methods

### Participants

The research team and the clinical team are working closely together within the framework of the Eating Disorders Treatment and Research Center at the University hospital. Patients acutely ill with anorexia nervosa (AN) (and their guardians) were contacted directly after admission to the eating disorder treatment programs. At the end of treatment patients participated in a follow up research assessment if they had successfully accomplished a body-mass index (BMI) increase of at least 14%, leading to approx. 70% inclusions relative to participants at admission. Patients recovered from AN were recruited through local and national advertising and by re-contacting participants who had participated (or had expressed willingness to participate) when they were ill with AN. Eligibility for inclusion in the study was determined by an expert clinician after consideration of medical and psychiatric history and diagnostic instruments such as the semi-structured interview SIAB-EX (1) and the Mini-International Neuropsychiatric Interview for Children and Adolescents (MINI Kid) (2), carried out by clinically experienced and trained research assistants under the supervision of a trained child and adolescent psychiatrist. Approx. 75% of participants screened met the inclusion criteria.

### Diagnostic procedures, inclusion and exclusion criteria

The diagnostic procedures, inclusion and exclusion criteria of the current study were identical to those in our previous sMRI studies (3–6). Of the present 165 scans of acutely underweight patients with AN (acAN-TP1), 115 scans of patients after partial weight-restoration (acAN-TP2), 89 scans of former patients after weight-recovery (recAN), and 289 scans of healthy controls (HC), 111, 81, 56, and 183 scans, respectively, were included in our previous study (3). This corresponds to additional 54 acAN-TP1, 34 acAN-TP2, 34 recAN, and 106 HC brain scans in the present study.

Information on inclusion and exclusion criteria (including possible confounding variables such as menstrual cycle and contraceptive use) was gained from all participants using the SIAB-EX (1), and for recAN and HC additionally the (MINI Kid) (2), carried out by clinically experienced and trained research assistants under the supervision of a trained child and adolescent psychiatrist. The interview systematically evaluates the prevalence and severity of the specific eating related psychopathology and provides diagnoses according to ICD-10 and DSM-V including a determination of AN subtype (Table 1).

Anthropometric data included height and weight measurement directly before scanning, from which we calculated both BMI and BMI-SDS [as gauged by age-adjusted BMI standard deviation scores (BMI-SDS) calculated according to (7) and German population reference data (8,9)] as outlined in the main Method section. Socio-economic status (SES) was determined according to the parental (household) educational level/occupation group [range: 0 (lowest), leaving school without graduation – 5 (highest), graduating from university] (10), given that most study participants were adolescent, current students at school, university, or professional training institutions, and still lived with their parents or guardians.

HC were excluded if they had any history of psychiatric illness, a lifetime BMI below the 10^th^ age percentile (if younger than 18 years)/BMI below 18.5 kg/m² (if older than 18 years) or were currently obese (BMI over 95^th^ age percentile if younger than 18 years; BMI over 29 kg/m² if older than 18 years). Participants of all groups were excluded if they had a history of any of the following diagnoses: organic brain syndrome, schizophrenia, substance dependence, psychosis not otherwise specified (NOS), bipolar disorder, bulimia nervosa, or binge-eating disorder. Further exclusion criteria for all participants were intelligence quotient (IQ) < 85, psychotropic medication within 4 weeks prior to the study (apart from selective serotonin reuptake inhibitors for which we had a more lenient criterium of two weeks prior to study participation which was not fulfilled by only 4 acAN-TP1), current substance abuse, inflammatory, neurologic or metabolic illness, chronic medical or neurological illness that could affect appetite, eating behavior, or body weight (e.g. Diabetes), clinically relevant anemia, pregnancy or breast feeding.

Comorbid psychiatric diagnoses were made in patients with AN by an expert clinician leading the treatment team and included examinations of the patients, discussions with the treatment staff and careful chart review (including consideration of medical and psychiatric history, physical examination, routine blood tests, urine analysis and several psychiatric screening instruments). All AN groups included some participants with at least one comorbid condition (acAN-T1 N=28, acAN-TP2 N=14, recAN N=37). The most common diagnoses were depressive disorders (acAN-TP1 N=13, acAN-TP2 N=6, recAN=27), anxiety disorders (acAN-TP1 N=10, acAN-TP2 N=5, recAN N=5), and obsessive-compulsive disorders (acAN-TP1 N=6, acAN-TP2 N=2, recAN N=2).

Demographic and clinical data were managed using a web-based tool [Research Electronic Data Capture (REDCap); <http://www.project-redcap.org>; (11)].

### Refeeding intervention

In the admission interview, patients with anorexia nervosa (AN) and their guardians were interviewed regarding the patient’s diet, caloric intake and body weight development in the past weeks. On the basis of this information alongside objective clinical measures (severity of weight loss and current body weight/BMI, somatic complications, risk factors for refeeding syndrome), a starting daily caloric intake was determined by an experienced child and adolescent psychiatrist (senior author). Intensive inpatient refeeding as defined by Bargiacchi et al. (12) (starting daily caloric intake 1500-2400 kcal/day, expected rates of body weight gain 0.5-2 kg/week) was achieved completely orally (none of the patients in our sample was refed with a nasogastric tube). Patients followed a structured meal plan (three meals, three snacks, fluid intake of 2 liters/day), developed in collaboration with a nutritionist, which was individually adjusted in regard to daily caloric intake. Body weight was monitored daily and caloric intake was typically increased every two to three days, depending on individual body weight gain. Hydration status was carefully monitored by regularly examining patients regarding skin turgor, edema, blood pressure and pulse.

While most patients quickly assimilated to the inpatient setting and started eating all served meals on the first or second day of inpatient treatment, a few patients refused to eat individual meals or food items. In this case, patients were encouraged to drink a corresponding quantity of a high-calorie nutrition product (Fresubin Energy Fibre Drink).

AcAN assessed at the second time-point (acAN-TP2) had achieved the requirements to be discharged from in-hospital treatment, which included reaching a BMI comparable to their normal weight and maintaining it for one week. In practice acAN-TP2 had increased their BMI by at least 14% and were scanned ~3 months on average after their acAN-TP1 appointment.

### Clinical measures

We assessed eating disorder-related psychopathology with the Eating Disorders Inventory [EDI-2, (13)] and affective symptoms with the Beck Depression Inventory [BDI-II, (14)] within one week of scan time. For acAN patients, treatment outcome one year after admission was assessed using the Morgan-Russell assessment schedule (15) which was performed by trained interviewers and supervised by an experienced psychologist. Due to the young age of many participants, we calculated the mean score of the subscales ‘food intake’ (to which BMI measured at follow-up contributes), ‘menstrual state’, ‘mental state’, and ‘socioeconomic state’ and excluded the subscale ‘psycho-sexual state’ as in one of our previous works (16).

### MRI processing and quality control of sMRI images

All participants underwent MRI scanning between 8 and 9 a.m. following an overnight fast. High resolution 3D T1-weighted structural scans were acquired on a 3.0T scanner (Magnetom Trio, Siemens, Erlangen, Germany) using a rapid acquisition gradient echo (MP-RAGE) sequence with the following parameters: 176 sagittal slices (1 mm thickness, no gap), TR = 1900 ms; TE = 2.26 ms; flip angle = 9˚; voxel size = 1.0 × 1.0× 1.0 mm, FoV = 256 × 224 mm^2^, bandwidth of 200 Hz/pixel).

Raw images were processed using standard FreeSurfer procedures (<http://surfer.nmr.mgh.harvard.edu/>; version 7.1) to achieve a sub-millimeter reconstruction of the cerebral cortex. The technical details of these procedures are described in prior publications (17–25). In sum, images underwent motion correction, skull stripping, automated Talairach transformation, segmentation of the subcortical white matter and deep gray matter volumetric structures, intensity normalization, tessellation of the gray matter white matter boundary, automated topology correction, and surface deformation following intensity gradients to optimally place the gray/white and gray/cerebrospinal fluid borders at the location where the greatest shift in intensity defines the transition to the other tissue class. The surface obtained can be inflated and registered to a spherical atlas which is based on individual location of main sulci and gyri. This allows comparison of cortical geometry across participants and parcellation of the cerebral cortex into units with respect to gyral and sulcal structure.

The quality of the surface reconstruction and segmentation was assessed following the ENIGMA Cortical Quality Control Protocol 2.0 (http://enigma.ini.usc.edu/protocols/imaging-protocols/). Visual inspection by a trained examiner was supported by quality assurance tools implemented in FreeSurfer (<https://surfer.nmr.mgh.harvard.edu/fswiki/QATools>) and exploratory analysis of the parcellation and segmentation statistics for outliers. Scans for which at least three of these measures deviated by more than 2.698 standard deviations relative to the group mean were inspected more closely by a trained rater using Freeview. If artifacts significantly affecting parcellation were detected, like inclusion of skull tissue in grey matter, obliterations in Talairach view, ghosting or spikes in Talairach view, the scan was excluded from the analysis.

### Nonlinear classifier

To assess whether non-linear patterns of brain structural alterations were associated with AN, we tested whether using a non-linear classifier could substantially improve the classification performance. Specifically, the support vector machine (SVM) was substituted in the classification pipeline by a neural network with one hidden layer. We explored a hidden layer with sizes of two to four neurons, different activation functions (sigmoid, tanh, ReLU), and L2 regularization values from 0.0001 to 30. Considerably more neurons, additional hidden layers, or weaker regularization increase the model complexity too much and thus produced strong overfitting judging from learning curves. While these comparably low numbers of hidden neurons only produce a weak non-linear extension, it was shown that any decision boundary can be approximated by a continuous feed-forward neural network with only a single internal hidden layer (26). We also tested other non-linear classification algorithms (kernel SVM, Random Forest), but found them all to not exceed the linear SVM performance. Thus, they were not considered further.

### Hyperparameter optimization

Our classification pipelines had free hyper-parameters. For example, in our linear classification pipeline the number of PCA components and the SVM regularization constant $C$ were free hyperparameters Therefore, our model selection procedure included both optimization of the model parameters (which we refer to as training) and of the hyperparameters.

To optimize the hyperparameters we performed grid search with and cross-validation (five times repeated, stratified to maintain the proportion of cases and controls equal across folds, ten-fold). We used PR-AUC as optimization metric. Compared to the more traditional metric accuracy, this metric captures the trade-off between positive-predictive-value/precision and sensitivity/recall in an unbalanced dataset.

In cross-validation, the dataset is partitioned into the given number of folds to repeatedly aggregate training/validation splits of the data. In each split, a different fold is held out and the model is trained on the remaining folds (training set). Subsequently, the held-out fold (validation set) is used to assess the performance of the trained model. In the context of grid search, this procedure is repeated for each combination of the hyperparameters. The best hyperparameters were selected as those optimizing performance on the validation set, measured as the average across splits. For further details on the grid values and results, see Table S2.

Finally, a classifier with the thus found optimal hyperparameters is retrained on the whole dataset used within cross-validated grid search to obtain a concrete model which can be used for inference on new unseen data.

### Estimation of model performance

To estimate the performance of the classifiers obtained using the above-mentioned model selection procedure (optimization of the model parameters and hyperparameters) we applied nested cross-validation (Figure S2). In nested cross-validation, the above described model selection via cross-validation was embedded in an outer cross-validation loop (ten times repeated, stratified, and ten-fold). As a result, in each split the entire dataset is divided in three: (i) the test set (one outer fold), used to assess the performance of the model selection procedure, (ii) the validation set (one inner fold), used to optimize the hyperparameters, and (iii) the training set (remaining inner folds), used to optimize the model parameters. The aim of nested cross-validation is to estimate performance on data that was not used (the test set), neither for training, nor for optimization of the hyperparameters. Specifically, for each split in the outer test loop the performance of the model with optimal hyperparameters (as determined based on the inner validation loop) was then assessed on the test set that had not been used so far (neither for training, nor for optimization of the hyperparameters). Thus, for each outer cross-validation split two performance measures (and corresponding PR/ROC curves) were available: (i) the test performance, which was computed on the test set, and (ii) the validation performance, which was computed on the validation set using the procedure described above. The validation performance might be an optimistically biased performance estimate of the model due to information leakage caused by optimizing hyperparameters on these validation sets. Therefore, the final measure of model performance was obtained from the average test performance (Figure 2, Figure S2). The latter is the average over all outer cross-validation splits, where the model with optimal hyperparameters is retrained on the outer training set and its performance is evaluated on the so far unseen test set. Since we split a complete dataset (HC/acAN-TP1: N=454, HC/acAN-TP2: N=404, HC/recAN: N=379) into ten folds, each outer training set contained 90% of the full dataset (acAN-TP1: N=410, acAN-TP2: N=364, recAN: N=341) and each test set contained the remaining 10% (acAN-TP1: N=44, acAN-TP2: N=40, recAN: N=38).

The difference between the test and validation performance (commonly called generalization gap) can inform whether the model obtained (with optimal parameters and hyperparameters) will generalize well to unseen data. This is the case if the generalization gap is small. Conversely, a large generalization gap is an indication that the model is overfitting the training and validation data.

### Permutation tests: model performance

To test whether the trained classifiers performed significantly better than chance, we employed permutation tests (27,28) as described by (29).

To construct the null distribution of performances for our model fitting procedure we first determined the optimal hyperparameters on the whole dataset using cross-validation as described above. For each permutation of the labels (i.e. whether the scan belonged to a participant with a history of AN or a HC) we computed AUC as an average over cross-validation splits (stratified, ten-fold). This procedure was repeated 100000 times. Finally, the probability to obtain a performance equal or higher than the one estimated with nested cross-validation was computed, based on the empirical null distribution of permuted AUCs obtained.

### Permutation tests: role of confounding variables

Since classifiers are highly sensitive to any kind of exploitable group differences in the data, we also assessed to which extent the information used for classification originated from confounding variables as opposed to sMRI measures, by applying the post-hoc method proposed by Dinga et al. (30). In short, the method aims to establish, after the machine learning model is fitted, the fraction of deviance explained by: (i) exclusively machine learning predictions (ii) exclusively confounds, and (iii) both machine learning predictions and confounds. Deviance is a goodness-of-fit statistic analogous to explained variance but for categorical variables, such as the labels that indicate group membership (acAN-TP1, acAN-TP2, recAN, HC). The machine learning-based risk scores of the classifier trained on the whole dataset were taken as the machine learning predictions. For each data point, they corresponded to the signed distance from the separating hyperplane for SVMs, or the probability output for neural networks. Similarly to above, permutation tests were used to assess whether fractions of deviance were significantly bigger than zero. Importantly, in this case only sMRI features were permuted relative to the group variable but confounds remained unpermuted (30,31). As above, for each permutation we computed the fractions of deviance explained as an average over cross-validation splits (stratified, ten-fold).

### Long-term outcome prediction

For acAN-TP2 who completed the one-year post-admission Morgan-Russel interview (N=74), we tested whether eventual multivariate structural alterations were related to long-term treatment outcome. To this end, we built GLMs with Morgan-Russel outcome score as dependent variable.

The baseline GLM had the machine learning-based risk score as independent variable and no additional covariate. In addition to the machine learning-based risk score, the other GLMs considered had (i) current BMI-SDS, or (ii) BMI-SDS increase since admission to treatment, or (iii) comorbidity at admission as additional covariate. The binary variable comorbidity indicates the presence or absence of a comorbid disorder at admission to the treatment program. Due to the low number of specific diagnoses we only considered this coarse differentiation. Of 14 acAN-TP2 patients with any comorbidity, the most frequent diagnoses were depressive (N=6) and anxiety (N=5) disorders. The subsample of acAN-TP2 patients for whom the Morgan-Russel outcome was available did not differ significantly in number of diagnosed comorbid conditions from those for whom this information was not available (Table S1). Moreover, long-term Morgan-Russel outcome was not significantly worse in acAN-TP2 patients with any comorbidity compared to those without comorbidity (one-sided Mann-Whitney U test p=0.64).

For GLM regression coefficients, we reported significance levels corresponding to one one-tailed tests, reflecting our hypothesis that more pronounced multivariate alterations in acAN-TP2 indicate worse long-term outcome. The Morgan-Russel outcome target distribution showed left-skewness and was therefore exponential transformed. The free parameter of the transformation was set to optimize the Gaussian shape of the resulting regression residuals, assessed via the Kolmogorov-Smirnov test.

### Feature importance and reliability

In order to evaluate the extent to which each feature impacted the classifier's decision, we utilized the method of feature importance estimation as outlined in Haufe et al. (32). For our specific scenario of binary classification with a linear model, this involved calculating the Pearson correlation coefficients between the classifier's machine learning-based risk score and each feature. For features with positive importance, a higher value indicates a higher likelihood of being classified in the AN group. Conversely, for features with negative importance, a lower value indicates a higher likelihood of being classified in the AN group. To determine if the feature importances were statistically significant, we computed the Bonferroni-corrected p-values of the Pearson correlation coefficients. To further evaluate the reliability of each feature, we performed two actions: (i) repeating the training of the model using various subsets of the complete dataset, and (ii) determining the proportion of subsets where a feature had an importance value that was statistically significant (33). We considered 500 subsamplings, each containing 90% of the full dataset, and maintaining the same class ratio as the full dataset.

To improve the interpretability of our main feature importance results we additionally computed feature importance with the method of permutation importance (34), as these two notions of feature importance may provide complementary insights. Permutation importance captures the amount of unique information that a feature provides for classification. Specifically, it ignores information useful for classification that is also present in other (typically correlated) features. Thus, the permutation importance method would not consider a feature to be important if another correlated feature is present, even if the feature contains information useful for classification. In contrast, the method by Haufe et al. considers a feature to be important if it contains information useful for classification, regardless of whether another feature might contain similar information. This is advantageous, since it provides an overarching description of the alterations that allow a classification. To obtain permutation importance values one computes the average decrease in performance when repeatedly permuting a given feature, i.e. eliminating its unique information. Obtained importance values range from zero (no unique information) to higher positive values (high importance). The highest possible importance value is given by the difference between model performance (unpermuted features) and chance performance. This would correspond to the edge case of a model which relies only on one feature which provides all the (unique) information, whereas all other features are uninformative. We utilized the ‘permutation_importance’ function of the scikit-learn python library with 500 permutations per feature.

### ENIGMA Toolbox

To provide macroscale network contextualization of feature importance profiles, we relied on the ENIGMA Toolbox (35). Normative connectivity matrices according to the Desikan-Killiany parcellation were extracted using healthy adults data from the HCP (36). Functional connectivity matrices were based on pairwise correlations between the time series of all cortical regions, where negative connections were set to zero. Participant-specific connectivity matrices were then z-transformed and aggregated across participants to construct a group-average functional connectome. No threshold was applied. Structural connectivity matrices were generated from preprocessed diffusion MRI data. Subsequently, network degree centrality maps were derived from functional (or structural) connectivity matrices by computing the weighted sum of all connections for every cortical region. Hubs denote those regions with higher network centrality, i.e. regions corresponding to network nodes with a high number of ties. To account for the intrinsic spatial smoothness in cortical maps, significance was tested using spin permutation tests (37) with 100000 permutations.

### Transfer classification

To get insights as to whether the fitted classifiers based their decisions on state as opposed to trait markers, we used a model trained to classify AN vs. HC at one time point (e.g. acAN-TP1 vs. HC) and applied it to classify at another time point (e.g. acAN-TP2 vs. HC) (38). To this end, the hyperparameters were set to their optimal values for the training time point data (e.g. acAN-TP1 vs. HC, see Table S2).

Of note, since for our participants with a history of AN MRI scans taken at different timepoints were used in the analysis (Figure S1), it was necessary to ensure that if a scan belonging to a given participant was used in the training set, no scan belonging to the same participant was used in the test set when assessing the performance of the transfer classification. Since the overlap for the acAN-TP1 to acAN-TP2 was substantial (Figure S1), we implemented a ten-fold cross-validation scheme to keep a high number of participants in each training and test set.

Specifically, for each split the model was trained on 90% of the scans available from the first time point (e.g. acAN-TP1) and applied to $\sim$10% of the scans available from the second time point (e.g. acAN-TP2), with the constraint that the latter scans belonged to different participants. Furthermore, when participants with AN, already included in the training set of one time point were dropped from the test set of the other time point the class ratio in the test set was re-adjusted to that of the training set by subsampling the HC participants in the test set accordingly. For example, the training set might contain a ratio of 1:2 between acAN-TP1 and HC. But the test set might contain more than double the amount of HC than acAN-TP2 since some AN already were included in the training set with an acAN-TP1 scan and thus were dropped from the test set. Then HC were randomly dropped from the test set to retain the 1:2 ratio. The estimated performance resulted as a weighted average of the AUC values across the splits where the weights were set based on the sample size of each test set (Figure S13).

## Supplementary results

### Hyperparameter optimization and model performance

The results of the hyperparameter optimization procedure are reported in Table S2 and the performance of the SVMs estimated through nested cross-validation is displayed in Figure S3. Similar performance on the validation and test samples indicated that our model selection procedure did not introduce a substantial amount of hyperparameter overfitting.

### Nonlinear classifier

The evaluation metrics (Figure S5) show that the neural network performs very similarly to the SVM discussed in the main manuscript, for each of the classification tasks. Specifically, it also does not classify significantly beyond confounds in the recAN classification task. Furthermore, both the SVM (Figure 3) and the neural network (Figure S9) identify very similar multivariate patterns in all classification tasks where performance above chance was achieved, as indicated by strong correlations between feature importance values (Spearman’s $\rho_{acAN-TP1}=0.99, p<0.001$ and $\rho_{acAN-TP2}=0.89, p<0.001$).

### Permutation importance

Additionally to our main results on feature importance we also computed feature importance with the method of permutation importance to determine which of the important features (according to Haufe et al.) in fact provide unique information that is not found in any other feature.

We found that among the most important features in Figure 3B, only a few with high positive importance according to Haufe et al. also showed a relatively high permutation importance (right lateral orbitofrontal, left and right insula cortical thickness), while all others (including several cortical regions with negative importance and some ventricular volumes) showed low permutation importance (Figure S10). This finding is in line with our interpretation that higher cortical thickness in the orbitofrontal and insula regions may constitute distinguished and regionally specific alterations in AN, while the latter group of features may reflect a residual of the widespread reduction in gray matter due to undernutrition.

### Adolescent subsample analysis

Our acAN-TP2 sample included mostly adolescent participants (83% of acAN-TP2 patients were younger than 18 years). We repeated our main analyses in a subsample comprising only adolescent participants from the acAN-TP2 (N=95) and HC (N=152) group. A machine learning analysis for the adult subsample was unfeasible due to the low number of acAN-TP2 patients older than 18 years (N=20). We trained the machine learning model on the adolescent subsample using the same hyperparameters as for the full sample (since for this reduced sample size, hyperparameter optimization as described in supplementary methods 1.4 resulted in considerable overfitting). The feature importances obtained for the adolescent subsample were highly correlated (Pearson correlation r=0.93) with those for the full sample (Figure 3) and the machine learning based risk score obtained based on the adolescent subsample was still significantly associated with Morgan-Russel outcome (N=63, p=0.008). As in the full sample, this remained true when the covariates of BMI-SDS (p=0.008), BMI-SDS change since admission to treatment (p=0.01), or psychiatric comorbidity (p=0.01) were included.

## Tables

| **Table S1.** Group comparison of acAN-TP2 with and without Morgan-Russel outcome score. | | |
| --- | --- | --- |
|  | **t** | **p** |
| **Machine learning-based risk score acAN-TP2** | -1.654 | 0.101 |
| **Age** | 0.207 | 0.836 |
| **EDI-2** | 2.764 | 0.007 |
| **BDI-2** | 3.579 | 0.001 |
| **BMI-SDS** | 0.346 | 0.730 |
| **EDI-2 (at admission)** | 0.924 | 0.358 |
| **BDI-2 (at admission)** | 2.054 | 0.042 |
| **BMI-SDS (at admission)** | -1.008 | 0.316 |
| **Comorbidity (at admission)** | 0.756 | 0.385 |

Group comparison between acAN-TP2 with (N=74) and without (N=41) Morgan-Russel outcome score. Continuous variables with skewed distributions were log/exp transformed before testing. Comorbidity indicates the presence of comorbid disorders at admission to treatment (Supplementary methods 1.8). Both samples generally had comparable measures, but acAN-TP2 with Morgan-Russel outcome score had higher EDI-2 and BDI-2 scores after treatment and higher BDI2 before treatment.

**Table S2.** Optimal hyperparameters found with grid search.

| **Classification task** | **PCA %Variance** | **SVM** $\boldsymbol{C}$ |
| --- | --- | --- |
| **acAN-TP1 vs. HC** | 0.7 | 0.0001 |
| **acAN-TP2 vs. HC** | 0.7 | 0.05 |
| **recAN vs. HC** | 0.75 | 0.005 |

For every AN time point vs. HC dataset a linear SVM model pipeline was built that included confounds subtraction and principal component analysis (PCA) for feature reduction. Two parameters had to be optimized, using grid search and cross-validation: The percent of variance explained by the retained principal components (PCA %Variance), and the SVM regularization parameter (SVM $C$). The searched grid values were PCA %Variance = [0.5, 0.6, 0.65, 0.7, 0.75, 0.8, 0.85, 0.9, 0.95, 1.0] and SVM $C$ = [1e-4, 5e-4, 1e-3, 5e-3, 1e-2, 5e-2, 0.1, 0.5, 1.0, 10.0, 50.0]. Applying PCA with the displayed percentage of retained variance to the whole datasets resulted in a number of retained components of 17 (acAN-TP1), 23 (acAN-TP2), and 23 (recAN). Importantly, the number of events per variable (EPV) after performing PCA was >13 for every classification task.

## Figures


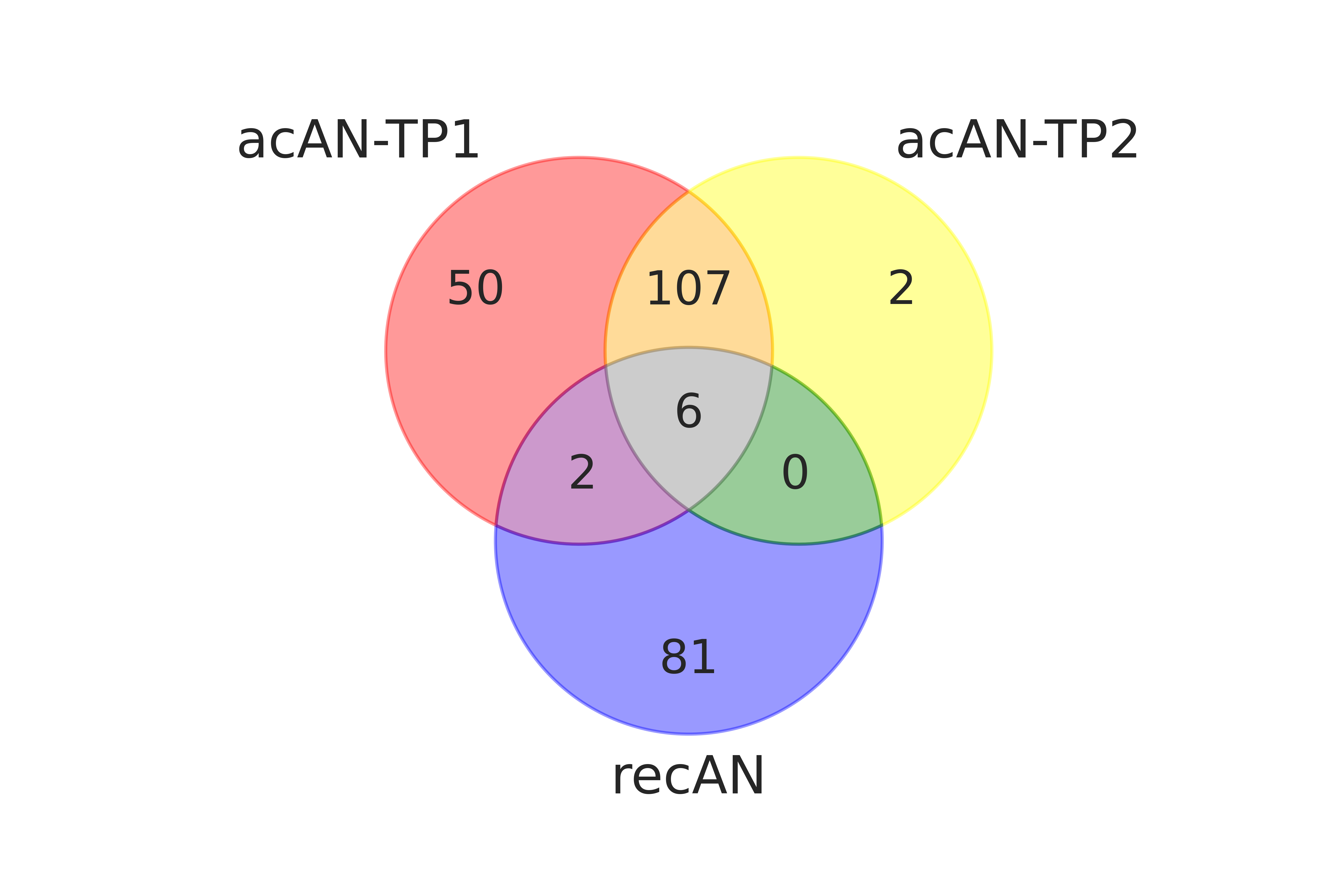


**Figure S1.** Overlaps of AN structural scan samples. Of all participants in the final samples 50 were solely included as acAN-TP1, two solely as acAN-TP2, and 81 solely as recAN. For 107 acAN a scan was included for acAN-TP1 and acAN-TP2, respectively. Two participants were included as acAN-TP1 and recAN. For six participants, a scan for every of the three time points was included, respectively.


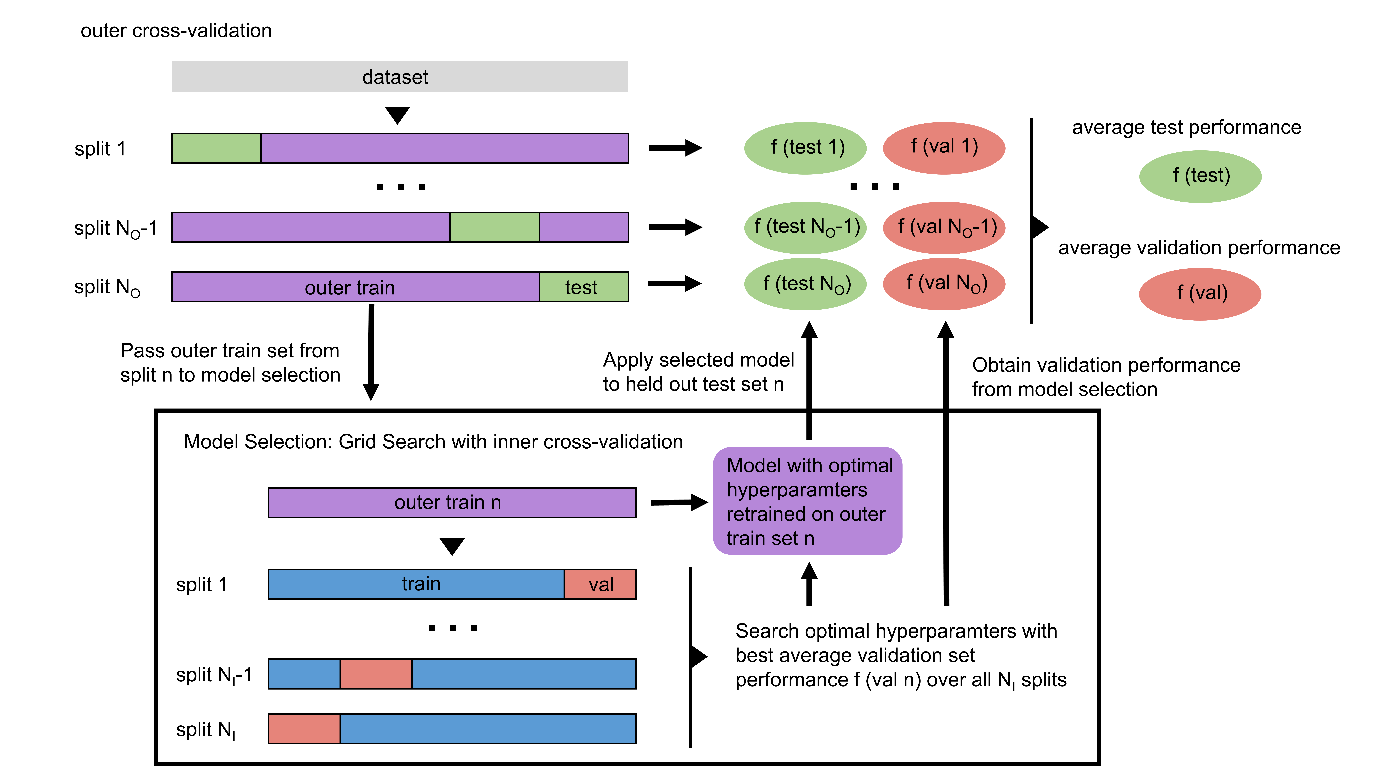


**Figure S2.** Nested cross-validation (Supplementary methods 1.5). This procedure is used to estimate unbiased model performance when both parameters and hyperparameters need to be fitted. An outer cross-validation loop splits the whole dataset into outer training and test set N_O_ times. For each split, the outer training set (outer train) is passed to the model selection procedure to search for the optimal model hyperparameters (Supplementary methods 1.4). This involves an inner cross-validation loop which splits the outer training set into training (train) and validation (val) set N_I_ times. The optimal hyperparameters maximize the average validation set performance f (val, n). Subsequently, for each outer split, the model with optimal hyperparameters is retrained on the outer training set and its performance f (test, n) is evaluated on the so far unseen test set. Finally, averages over the N_O_ outer splits give the test performance estimate f (test), and the validation performance f (val). f (test) constitutes our final estimate of the model performance. f (val) would be a biased estimate (over-optimistic). A large gap [f (val) >> f (test)] would indicate that the model is overfitting the data.





**Figure S3.** SVM test and validation performance curves estimated via nested cross-validation for acAN-TP1, acAN-TP2 and recAN vs. HC classifications (Supplementary methods 1.5). These are the same results as in Figure 2 of the main manuscript, with additional validation (val) curves. **A:** Precision-Recall-curves and **B:** corresponding ROC-curves. Shown are (ten times repeated, ten-fold) outer cross-validation averages and s.d. ranges. These test curves provide an estimate for the performance of our model selection procedure (Supplementary methods 1.4). Dashed lines represent chance performance. Permutation tests of the corresponding AUCs indicated a clear above-chance classification performance for acAN-TP1 and acAN-TP2 but not for recAN (Supplementary methods 1.6, Figure S4).


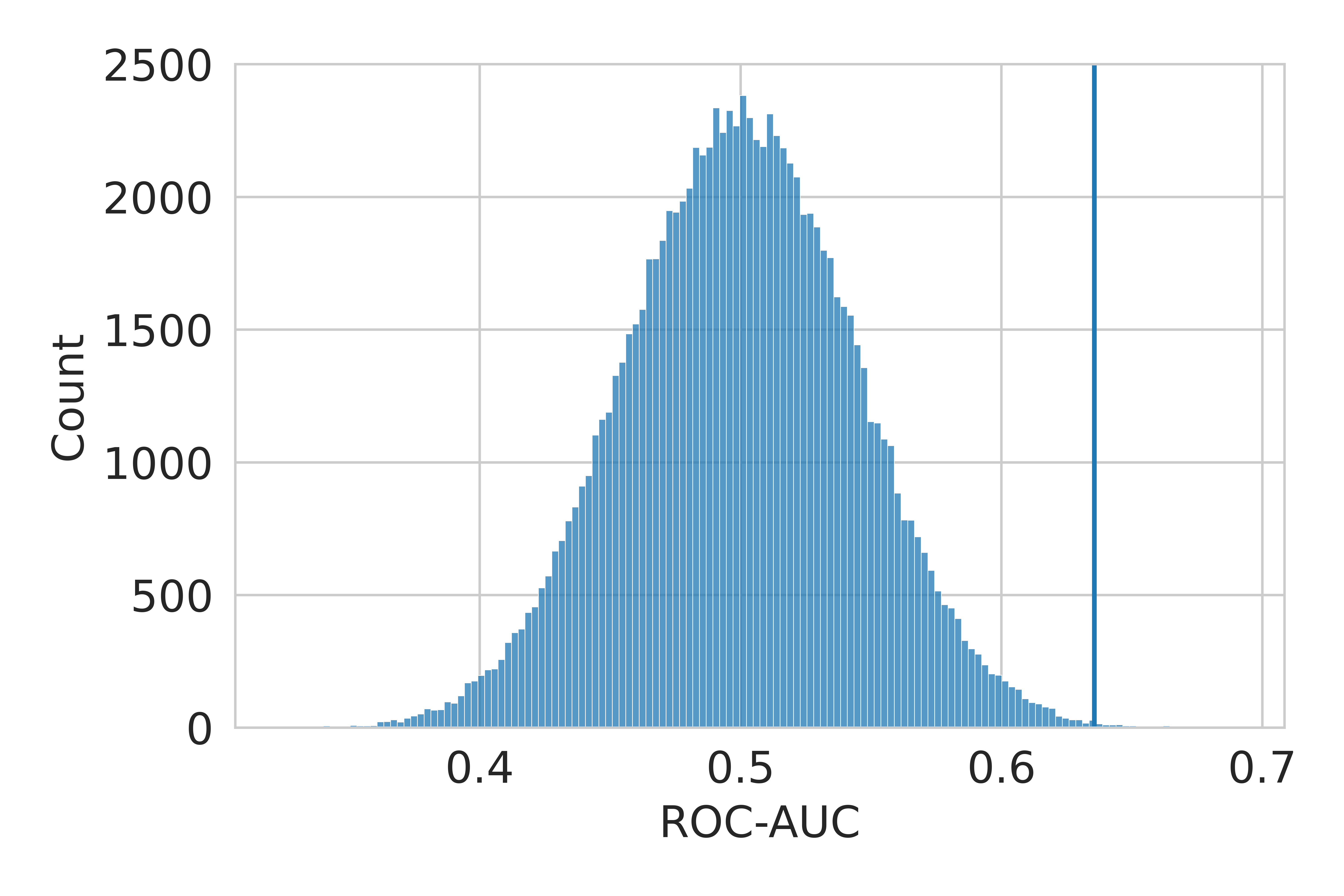


**Figure S4.**  Distribution of ROC-AUC permutation scores for the acAN-TP2 model with 100000 permutations. The solid line indicates the unpermuted score. The permutation test shows that the model significantly performed better than chance (p = 0.001).

**
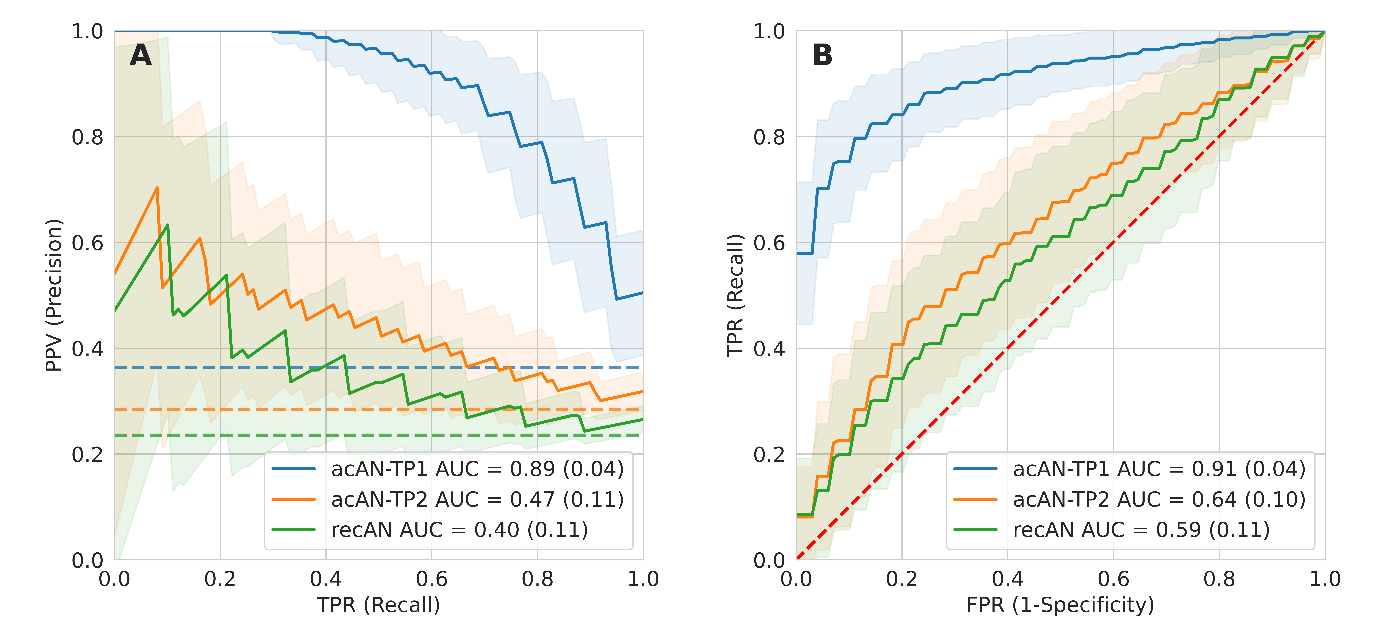
Figure S5.**  Neural network (Supplementary methods 1.3) validation performances for acAN-TP1 (blue), acAN-TP2 (yellow), and recAN (green) vs. HC classification do not exceed those of the SVM classifier in Figure 2 of the main manuscript. Shown are **A:** Precision-Recall-curves and **B:** corresponding ROC-curves averaged over ten repeats of ten-fold cross-validation and overlaid with the standard deviation range for models with the optimal hyperparameters obtained through a cross-validation procedure (Supplementary methods 1.3, 1.4). Dashed lines represent chance performance.





**Figure S6.** Importance values for features that significantly contributed to the acAN-TP1 vs. HC classification after Bonferroni correction for multiple comparisons. The color scale encodes reliability values. The only features that did not contribute significantly were the cortical thickness in both hemispheres of the transversetemporal, rostralanteriorcingulate, cautalanteriorcingulate, parahippocampal, entorhinal, and temporal pole (pole); in the left hemisphere of the insula and in the right hemisphere of the pericalcarine; and the volumes of the subcortical regions CC-mid-posterior, caudate, and CC-anterior volumes.


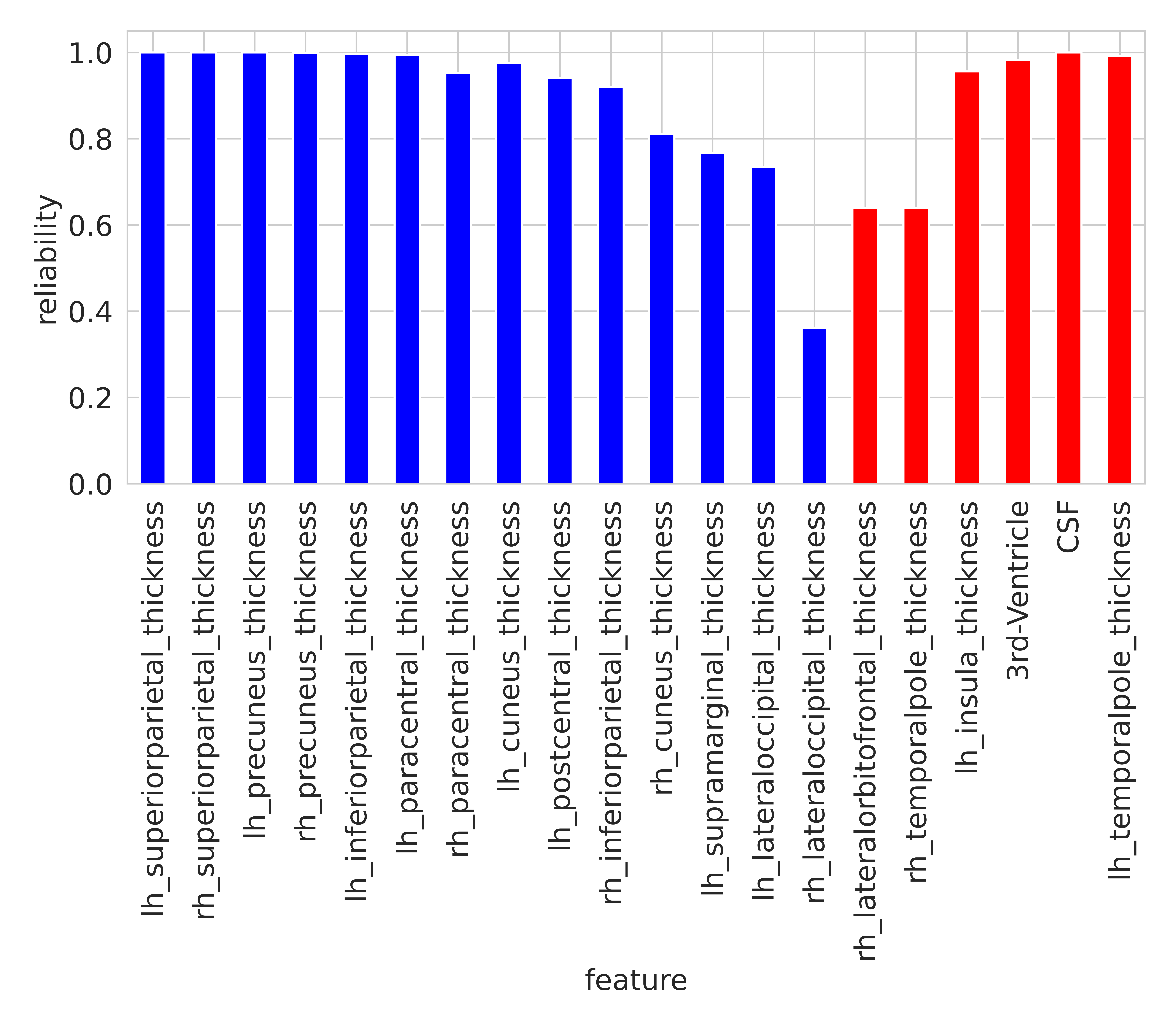


**Figure S7.** Reliability values for the acAN-TP2 vs HC classification. Blue/red indicates negatively/positively signed feature importance. Only features with significant Bonferroni corrected importance value are shown.





**Figure S8.** Reliability values for the acAN-TP1 vs. HC classification. Blue/red indicates negatively/positively signed feature importance. Only features with significant Bonferroni corrected importance value are shown.





**Figure S9.** Feature importance and reliability using a neural network instead of a SVM as classifier in the classification pipeline. **A:** Feature importance values for the acAN-TP1 model (x-axis) compared to the values for the acAN-TP2 model (y-axis). Measures for cortical thickness, volumes of subcortical grey matter regions, cerebrospinal fluid (CSF) spaces are shown in blue, red and orange, respectively. **B:** The ranking of feature importance values for the acAN-TP2 vs. HC classification strongly agreed with the one found for the SVM (Figure 3). The color code further illustrates the reliability values of features for classification. **C/D:** Feature importance values for the acAN-TP1/TP2 model plotted on the surface of the standard average brain (35). The color code illustrates the magnitude of negatively (blue) and positively (red) signed feature importance.

**
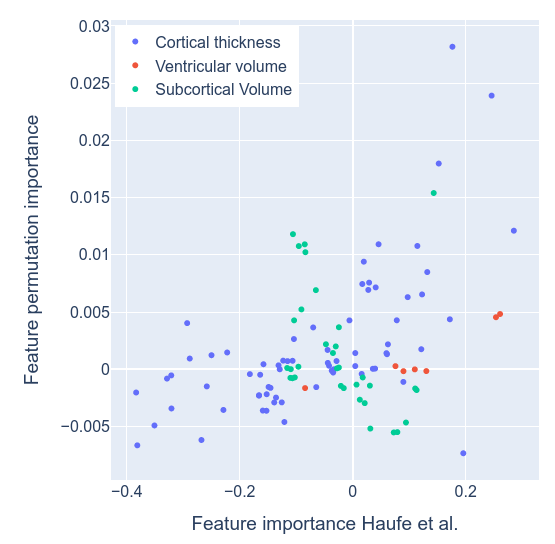
Figure S10.** Comparison between two different assessments of feature importance for the acAN-TP2 classification. Results obtained for permutation importance are plotted against those obtained using the definition proposed by Haufe et al. (x-axis). Values on the x-axis correspond to the results in Figure 3B of the main manuscript. Of note, since the two methods estimate different notions of feature importance, their absolute scales are not comparable. Permutation importance estimates the decrease in performance when eliminating the unique information provided by a feature, with values ranging from slightly negative/zero values (no unique information) to higher positive values (high importance). On the other hand, the feature importance method proposed by Haufe et al. estimates importance based on both shared and unique information, with the sign indicating whether high or low feature values are related to AN classification, respectively.





**Figure S11.** Correlations between network centrality (on the x-axes) and feature importance profiles (on the y-axes) of the linear SVM model for the acAN-TP2 vs. HC classification. Network degree centrality (hubs) was computed on functional **A, B:** and structural **C, D:** connectivity matrices. Correlations with feature importance values across the 68 cortical and 14 subcortical regions of the left and right hemispheres in the Desikan-Killiany atlas (18) are shown.





**Figure S12.** Correlations between network centrality (on the x-axes) and feature importance profiles (on the y-axes) of the linear SVM model for the acAN-TP1 vs. HC classification. Network degree centrality (hubs) was computed on functional **A, B:** and structural **C, D:** connectivity matrices. Correlations with feature importance values across the 68 cortical and 14 subcortical regions of the left and right hemispheres in the Desikan-Killiany atlas (18) are shown.





**Figure S13.** Cross-validated performances of SVM models with optimal hyperparameters (Table S2) for acAN-TP1/acAN-TP2 vs. HC classification, respectively (blue/orange), compared to performances achieved by the same models when classifying acAN-TP2/acAN-TP1 vs. HC, respectively (green/red). In the latter case we subsampled the over-represented class of the test data to ensure the same class ratio as in the training data. Shown are averages and the one standard deviation range over cross-validation splits (ten repeats, ten-fold) of **A:** PR-curves and **B:** corresponding ROC-curves with their AUCs. Dashed lines represent chance performance. While the acAN-TP2 model seemed to differentiate acAN-TP1 from HC (red) the acAN-TP1 model performance dropped to chance level when differentiating acAN-TP2 vs. HC (green). We also applied the models trained on acAN-TP1 or acAN-TP2 data to the recAN sample respectively but found them to perform at chance level.


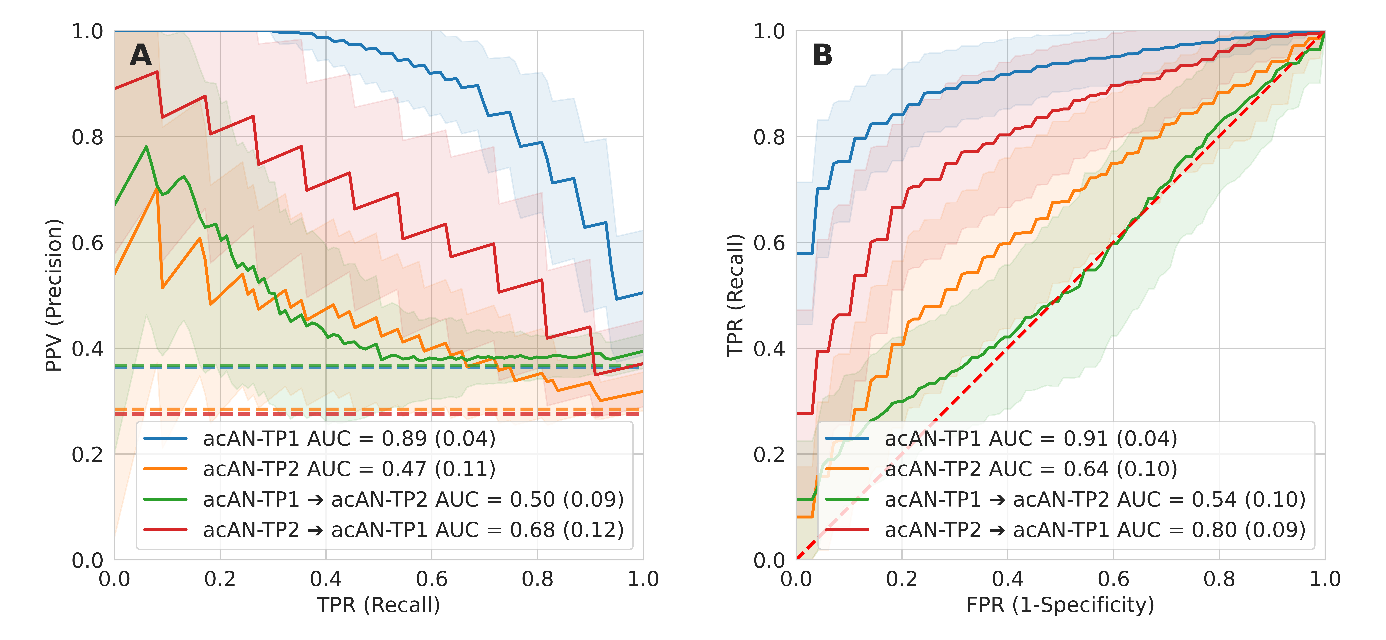


**Figure S14.** Validation performance curves for **A:** PR and **B:** ROC computed as in Figure S13 but for a non-linear neural network as classifier (Supplementary methods 1.3) in the model pipeline. The AUC performances did not exceed those of the linear SVM model.

## Supplementary references

1. Fichter M, Quadflieg N. The structured interview for anorexic and bulimic disorders for DSM-IV and ICD-10 (SIAB-EX): reliability and validity. Eur Psychiatry. 2001 Feb;16(1):38–48.

2. Sheehan DV, Lecrubier Y, Sheehan KH, Amorim P, Janavs J, Weiller E, et al. The Mini-International Neuropsychiatric Interview (M.I.N.I): The development and validation of a structured diagnostic psychiatric interview for DSM-IV and ICD-103. J Clin Psychiatry. 1998;59:22–33.

3. Bahnsen K, Bernardoni F, King JA, Geisler D, Weidner K, Roessner V, et al. Dynamic Structural Brain Changes in Anorexia Nervosa: A Replication Study, Mega-analysis, and Virtual Histology Approach. J Am Acad Child Adolesc Psychiatry [Internet]. 2022 Apr [cited 2022 Jun 29]; Available from: https://linkinghub.elsevier.com/retrieve/pii/S0890856722001836

4. Bernardoni F, King JA, Geisler D, Birkenstock J, Tam FI, Weidner K, et al. Nutritional Status Affects Cortical Folding: Lessons Learned From Anorexia Nervosa. Biol Psychiatry [Internet]. 2018 May [cited 2018 Sep 4]; Available from: https://linkinghub.elsevier.com/retrieve/pii/S0006322318315221

5. Bernardoni F, King JA, Geisler D, Stein E, Jaite C, Nätsch D, et al. Weight restoration therapy rapidly reverses cortical thinning in anorexia nervosa: A longitudinal study. NeuroImage. 2016 Apr 15;130:214–22.

6. King JA, Geisler D, Ritschel F, Boehm I, Seidel M, Roschinski B, et al. Global cortical thinning in acute anorexia nervosa normalizes following long-term weight restoration. Biol Psychiatry. 2015 Apr 1;77(7):624–32.

7. Cole TJ. The LMS method for constructing normalized growth standards. Eur J Clin Nutr. 1990 Jan;44(1):45–60.

8. Hemmelmann C, Brose S, Vens M, Hebebrand J, Ziegler A. [Percentiles of body mass index of 18-80-year-old German adults based on data from the Second National Nutrition Survey]. Dtsch Med Wochenschr 1946. 2010 Apr;135(17):848–52.

9. Kromeyer-Hauschild K, Wabitsch M, Kunze D, Geller F, Geiß HC, Hesse V, et al. Perzentile für den Body-mass-Index für das Kindes- und Jugendalter unter Heranziehung verschiedener deutscher Stichproben. Monatsschr Kinderheilkd. 2001 Aug;149(8):807–18.

10. Patrick K, Norman GJ, Calfas KJ, Sallis JF, Zabinski MF, Rupp J, et al. Diet, Physical Activity, and Sedentary Behaviors as Risk Factors for Overweight in Adolescence. Arch Pediatr Adolesc Med. 2004 Apr 1;158(4):385.

11. Harris PA, Taylor R, Thielke R, Payne J, Gonzalez N, Conde JG. Research electronic data capture (REDCap)—A metadata-driven methodology and workflow process for providing translational research informatics support. J Biomed Inform. 2009 Apr;42(2):377–81.

12. Bargiacchi A, Clarke J, Paulsen A, Leger J. Refeeding in anorexia nervosa. Eur J Pediatr. 2019 Mar;178(3):413–22.

13. Paul T, Thiel A. Eating Disorder Inventory-2 (EDI-2): deutsche Version. Hogrefe; 2005.

14. Hautzinger M, Keller F, Beck AT, Kühner C. Beck Depressions-Inventar : BDI II ; Manual. Pearson Assessment; 2009. 35 p.

15. Morgan HG, Hayward AE. Clinical Assessment of Anorexia Nervosa: The Morgan-Russell Outcome Assessment Schedule. Br J Psychiatry. 1988 Mar;152(3):367–71.

16. Boehm I, Mohr H, King JA, Steding J, Geisler D, Wronski ML, et al. Aberrant neural representation of food stimuli in women with acute anorexia nervosa predicts treatment outcome and is improved in weight restored individuals. Transl Psychiatry. 2021 Dec;11(1):532.

17. Dale AM, Fischl B, Sereno MI. Cortical surface-based analysis. I. Segmentation and surface reconstruction. NeuroImage. 1999 Feb;9(2):179–94.

18. Desikan RS, Ségonne F, Fischl B, Quinn BT, Dickerson BC, Blacker D, et al. An automated labeling system for subdividing the human cerebral cortex on MRI scans into gyral based regions of interest. NeuroImage. 2006 Jul 1;31(3):968–80.

19. Fischl B, Sereno MI, Dale AM. Cortical surface-based analysis. II: Inflation, flattening, and a surface-based coordinate system. NeuroImage. 1999 Feb;9(2):195–207.

20. Fischl B, Sereno MI, Tootell RB, Dale AM. High-resolution intersubject averaging and a coordinate system for the cortical surface. Hum Brain Mapp. 1999;8(4):272–84.

21. Fischl B, Salat DH, Busa E, Albert M, Dieterich M, Haselgrove C, et al. Whole brain segmentation: automated labeling of neuroanatomical structures in the human brain. Neuron. 2002 Jan 31;33(3):341–55.

22. Fischl B, Salat DH, van der Kouwe AJW, Makris N, Ségonne F, Quinn BT, et al. Sequence-independent segmentation of magnetic resonance images. NeuroImage. 2004;23 Suppl 1:S69-84.

23. Segonne F, Pacheco J, Fischl B. Geometrically Accurate Topology-Correction of Cortical Surfaces Using Nonseparating Loops. IEEE Trans Med Imaging. 2007 Apr;26(4):518–29.

24. Fischl B, van der Kouwe A, Destrieux C, Halgren E, Ségonne F, Salat DH, et al. Automatically parcellating the human cerebral cortex. Cereb Cortex N Y N 1991. 2004 Jan;14(1):11–22.

25. Ségonne F, Dale AM, Busa E, Glessner M, Salat D, Hahn HK, et al. A hybrid approach to the skull stripping problem in MRI. NeuroImage. 2004 Jul;22(3):1060–75.

26. Cybenko G. Approximation by superpositions of a sigmoidal function. Math Control Signals Syst. 1989 Dec;2(4):303–14.

27. Noirhomme Q, Lesenfants D, Gomez F, Soddu A, Schrouff J, Garraux G, et al. Biased binomial assessment of cross-validated estimation of classification accuracies illustrated in diagnosis predictions. NeuroImage Clin. 2014;4:687–94.

28. Golland P, Fischl B. Permutation Tests for Classification: Towards Statistical Significance in Image-Based Studies. In: Taylor C, Noble JA, editors. Information Processing in Medical Imaging [Internet]. Berlin, Heidelberg: Springer Berlin Heidelberg; 2003 [cited 2021 Mar 26]. p. 330–41. (Goos G, Hartmanis J, van Leeuwen J, editors. Lecture Notes in Computer Science; vol. 2732). Available from: http://link.springer.com/10.1007/978-3-540-45087-0_28

29. Ojala M, Garriga GC. Permutation tests for studying classifier performance. J Mach Learn Res. 2010;11(6).

30. Dinga R, Schmaal L, Penninx BWJH, Veltman DJ, Marquand AF. Controlling for effects of confounding variables on machine learning predictions [Internet]. Bioinformatics; 2020 Aug [cited 2021 Mar 8]. Available from: http://biorxiv.org/lookup/doi/10.1101/2020.08.17.255034

31. Höfling H, Tibshirani R. A study of pre-validation. Ann Appl Stat [Internet]. 2008 Jun 1 [cited 2021 Mar 26];2(2). Available from: https://projecteuclid.org/journals/annals-of-applied-statistics/volume-2/issue-2/A-study-of-pre-validation/10.1214/07-AOAS152.full

32. Haufe S, Meinecke F, Görgen K, Dähne S, Haynes JD, Blankertz B, et al. On the interpretation of weight vectors of linear models in multivariate neuroimaging. NeuroImage. 2014 Feb;87:96–110.

33. Nogueira S, Brown G. Measuring the Stability of Feature Selection. In: Frasconi P, Landwehr N, Manco G, Vreeken J, editors. Machine Learning and Knowledge Discovery in Databases [Internet]. Cham: Springer International Publishing; 2016 [cited 2021 Jun 15]. p. 442–57. (Lecture Notes in Computer Science; vol. 9852). Available from: http://link.springer.com/10.1007/978-3-319-46227-1_28

34. Breiman L. Random forests. Mach Learn. 2001;45:5–32.

35. Larivière S, Paquola C, Park B yong, Royer J, Wang Y, Benkarim O, et al. The ENIGMA Toolbox: multiscale neural contextualization of multisite neuroimaging datasets. Nat Methods. 2021 Jul;18(7):698–700.

36. Toga AW, Clark KA, Thompson PM, Shattuck DW, Van Horn JD. Mapping the Human Connectome: Neurosurgery. 2012 Jul;71(1):1–5.

37. Alexander-Bloch AF, Shou H, Liu S, Satterthwaite TD, Glahn DC, Shinohara RT, et al. On testing for spatial correspondence between maps of human brain structure and function. NeuroImage. 2018 Sep;178:540–51.

38. Zhang-James Y, Helminen EC, Liu J, Franke B, Hoogman M, Faraone SV. Evidence for similar structural brain anomalies in youth and adult attention-deficit/hyperactivity disorder: a machine learning analysis. Transl Psychiatry. 2021 Jun;11(1):82.
